# Overview of snakebite in Brazil: Possible drivers and a tool for risk mapping

**DOI:** 10.1371/journal.pntd.0009044

**Published:** 2021-01-29

**Authors:** Maria Cristina Schneider, Kyung-duk Min, Patricia Nájera Hamrick, Lucia R. Montebello, Tani Maria Ranieri, Lucia Mardini, Volney M. Camara, Ronir Raggio Luiz, Bernhard Liese, Myriam Vuckovic, Milton Ozorio Moraes, Nísia Trindade Lima

**Affiliations:** 1 Department of International Health, School of Nursing and Health Studies, Georgetown University, Washington, DC, USA; 2 Institute of Collective Health Studies, Federal University of Rio de Janeiro, RJ, Brazil; 3 Institute of Health and Environment, Graduate School of Public Health, Seoul National University, Seoul, South Korea; 4 Department of Health Emergencies, Pan American Health Organization, Washington, DC, USA; 5 Department of Immunization and Transmissible Diseases, Health Surveillance Secretary, Ministry of Health of Brazil, Brasilia, DF, Brazil; 6 Epidemiological Surveillance Division, State Health Department of Rio Grande do Sul, Porto Alegre, RS, Brazil; 7 Oswaldo Cruz Foundation (FIOCRUZ), Ministry of Health of Brazil, Rio de Janeiro, RJ, Brazil; Goethe University, GERMANY

## Abstract

Snakebite envenoming affects close to 2.7 million people globally every year. In Brazil, snakebites are reported to the Ministry of Health surveillance system and cases receive antivenom free of charge. There is an urgent need to identify higher risk areas for antivenom distribution, and to develop prevention activities. The objective of this study is to provide an overview of the epidemiological situation of snakebite envenoming in Brazil and explore possible drivers; as well as to create a flowchart tool to support decision-makers identify higher risk areas. An ecological-type study was carried out using data by municipality (2013–2017). Study parts: 1) Create a geocoded database and perform a descriptive and cluster analysis; 2) Statistical analysis to measure the association of snakebite and possible environmental and socioeconomic drivers; 3) Develop a flowchart to support decision-makers and the application of this tool in one state (Rio Grande do Sul) as an example. An average of 27,120 snakebite cases per year were reported at the country level. Clusters of municipalities with high numbers of snakebites are mostly found in the Amazon Legal Region. The negative binomial regression model showed association with the snakebite case count: the type of major habitat, tropical or non-tropical; temperature; percentage of urbanization; precipitation; elevation; GDP per capita; a weaker relation with forest loss; and with venomous snake richness. The state where the instrument was applied reported 4,227 snakebites in the period. Most municipalities were considered as medium risk and 56/496 as high risk according to the tool created. Snakebite cases are distributed across the entire country with the highest concentration in the Legal Amazon Region. This creates a complex situation both for better understanding of the association of environmental and socioeconomic factors with snakebites and for the distribution and maintenance of antivenom to remote areas. Research into types of antivenom with a longer shelf life without the need for refrigeration is needed.

## Author summary

It is estimated that snakebite envenoming affects around 2.7 million people every year worldwide. In Brazil, snakebite is part of the Ministry of Health surveillance system and cases receive antivenom free of charge. With a vast territory and limited production of antivenom, there is need to identify higher risk areas for its distribution and to develop preventative actions. The objective of this study is to provide an overview of the epidemiological situation of snakebite envenoming in Brazil and explore possible environmental and socioeconomic factors related to snakebites. On the basis of this information, a flowchart tool to support decision-makers identify higher risk areas was created and applied in one state as an example. An average of 27,120 snakebite cases per year were reported in Brazil, with the highest number of cases and rates in the Legal Amazon Region. The multivariable statistics analysis showed association of snakebite with environmental factors (type of major habitat, tropical or non-tropical; temperature; precipitation; elevation) and socioeconomic (lower percentage of urbanization; lower GDP per capita); also, a weaker relation with forest loss, and with venomous snake richness. The tool created to support decision-makers was applied in the state of Rio Grande do Sul, with 4,227 reported snakebites during the period. According to this tool, the majority of municipalities in this state were considered as medium risk and 56/496 as high risk. Snakebite cases are distributed across the entire country; however, the highest concentration is in the Legal Amazon Region. This creates a complex situation both for better understanding of the association of environmental and socioeconomic factors with snakebites and for the distribution and maintenance of antivenom to remote areas.

## Introduction

Snakebite envenoming is estimated to affect close to 2.7 million people globally every year, mostly in poor, rural communities in tropical and subtropical areas; it is also estimated that around 100,000 people annually die and another 400,000 suffer disability due to snakebite [1–3]. Antivenom has been available for 120 years and is highly effective, especially if administered early in an adequate dose [3]. However, the inattention to this public health issue and crises in antivenom production globally have left millions vulnerable, with lower chance of treatment after an envenoming accident to reduce the probability of death and disability.

Snakebite envenoming and death are usually associated with low socioeconomic status or poverty, and groups such as agricultural workers, indigenous populations, hunter-gatherers, herders, fishermen, rubber tappers, Brazilian nut extractors, families living in poorly constructed houses, and people with limited access to education and health care [4–7]. It is also considered an occupational problem in Brazil [8–10].

In 2017, the World Health Organization (WHO) included snakebite envenoming in its list of neglected tropical diseases and in 2019 its ‘Strategy for a globally coordinated response to a priority neglected tropical disease’ was approved [3, 7], with the aim of reducing mortality and disability from snakebite envenoming by 50% before 2030. Among the activities suggested is improving surveillance and data analysis [11].

Most snakebite cases occur in Africa, Asia and Latin America. Brazil, with its large forest areas and great variety of venomous snakes, is among the countries with the highest risk for envenomation by snakes. Brazil has a long tradition of reporting snakebites and producing antivenom, with the first study done by Vital Brazil in 1901 in the state of Sao Paulo, and antivenom production starting at the same time [4]. The Ministry of Health of Brazil implemented the National Program for Snakebite Control in 1986 and extended it to include other venomous animals in 1988 [6]. Envenoming accidents by animals such as snakes, scorpions, spiders, and others are subject to compulsory notification to the Ministry of Health of Brazil Notifiable Diseases Information System, National Health Surveillance Secretariat database (acronym in Portuguese SINAN) [12]. Currently, around 220,000 cases of accidents with venomous animals are officially reported in SINAN per year, including close to 30,000 cases of snakebite [12]. Antivenom and treatment is provided free of charge by the Brazilian health system [13].

Even though Brazil has been producing antivenom for more than 100 years, currently the amount available for government purchase is reduced due to restrictions in the productive capacity of the laboratories. Presently, there are four public laboratories in Brazil producing antivenom, which undergoes quality control before it is distributed to the states and onward to municipalities nationwide [14]. The Ministry of Health, since 1986, has acquired all the production of antivenom from the four national producers (Instituto Butantan, Instituto Vital Brazil, Ezequiel Dias Foundation, and Center for Production and Research of Immunobiologicals) [15]. Monthly, the Ministry of Health distributes antivenom quotas to the states, taking into account epidemiological criteria, which are notifications of accidents by venomous animals to SINAN. Currently, laboratories producing antivenoms in Brazil are in the process of adapting to the Good Manufacturing Practices of Brazil’s national drug regulatory agency (ANVISA), which is leading to an even more cautious distribution of antivenoms to the states [15].

According to the Ministry of Health of Brazil [13] the snakes of public health interest in the country belong to the Viperidae and Elapidae families. Antivenom is needed for snakebites involving five genera: *Bothrops*, *Bothrocophias*, *Crotalus*, *Lachesis*, and *Micrurus*. However, 72% of snakebite envenoming is caused by *Bothrops* [4, 13].

WHO [16] considers 278 snake species of medical importance globally, of which 23 are present in Brazil. To identify and estimate the populations most vulnerable to snakebite morbidity and mortality, Longbottom et al. [17] created maps based on the distribution of snakes of medical importance combined with other snake information and a statistical analysis with health system variables. According to that study, about 6.85 billion people worldwide live within range of snake habitats, and many of these areas are in Brazil. Other studies in Costa Rica and in Sri Lanka have also developed geo-information systems to analyze higher risk areas for snakebite [18, 19].

In Brazil, snakebite is a well-known public health problem and it is receiving attention from health authorities, including free of charge antivenom when the person is able to seek health care. Given the limited supply of antivenom worldwide and in Brazil, decision-makers need tools to help them best allocate antivenom, as well as to develop educational actions for prevention and training in medical care.

Considering the diversity in biomes in Brazil, the richness of snakes species, as well as the gaps in socioeconomic indicators across areas, we suggest that a One Health approach [20, 21] could be employed to improve understanding of the complexity of snakebite in Brazil, and to support decision-makers in their risk characterization.

The objective of this study is to provide an overview of the epidemiological situation of snakebite in Brazil and explore possible environmental and socioeconomic drivers; and based on this information, to create an instrument to support decision-makers to define higher risk areas for interventions and apply it in one state of Brazil (Rio Grande do Sul) as an example.

## Materials and methods

### Study overview

An ecological-type study was carried out using data aggregated by municipality to analyze the epidemiological situation of snakebite in Brazil between 2013 and 2017 by the second subnational administrative level (municipalities). For the purpose of this study snakebite envenomation will be referred as snakebite only. Brazil as a territory of 8,515,767.049 km² is divided in 26 states and one Federal District (first subnational level), and 5,570 municipalities at the second subnational level, ranging from 3.565 km² to 159,533.328 km². Although Brazil currently has a total of 5,570 municipalities, we used a previous categorization of administrative second subnational level, which consisted of 5,564 municipalities (Tables A and B in S1 Text).

The study has three parts: 1) a descriptive analysis of the epidemiological situation by state and by municipality using the major habitat type as background; a cluster analysis and selected thematic maps; 2) a statistical analysis to measure the association of snakebites and possible drivers; 3) the development of a flowchart to support decision-makers, and the application of this tool in one state as an example.

### Data sources and processing

The number of snakebite cases that received antivenom treatment reported to the Ministry of Health aggregated at the municipality level was obtained from the SINAN database [12]. All cases of snakebite that received antivenom treatment are reported to SINAN. This data is publicly available on the government website.

The selection of variables for the possible driver analysis was done based on previous studies on snakebite [17] and on infectious diseases in Brazil using geocoded variables [22–24].

A set of variables was created for the driver analysis: 1) Environmental: forest loss, temperature, precipitation, elevation, major habitat type, venomous snake richness, forest cover; 2) Socioeconomic: GDP per capita; 3) Demographic: percentage of urban population. These variables were either downloaded or created from original sources gathered from various open-access data sources (Tables A and B in S1 Text). Geo-processing techniques were applied to assign and measure environmental variables for each municipality (Tables A and B in S1 Text). A geocoded database was created from scratch.

The forest loss variable was acquired from the website provided by Hansen et al. [25] derived from satellite images obtained between 2000 and 2017 [26]. Hansen et al. used a classification algorithm that categorized the captured terrestrial surface into forest and non-forest regions and assessed forest presence in 2000 and annual deforestation levels from 2001 to 2017 (S1 Text). This was the same methodology applied in a previous publication [27, 28].

The variable major habitat type (biome categories) was obtained from FAO [29] Terrestrial Ecoregions of the World database and map with a bio-geographic regionalization of the Earth’s terrestrial biodiversity, and from WWF [30]. In the major habitat type map, the delimitation of the Legal Amazon Region of Brazil was also included, which shows similarity to the Tropical and Subtropical Moist Broadleaf Forests (TSMBF) major habitat.

The snake richness variable included the four venomous genera (*Bothrops*, *Crotalus*, *Micrurus*, and *Lachesis*) present in Brazil, according to WHO [31] and the Ministry of Health of Brazil [32]. The ten species included in this variable were those in the WHO list and repeated in the Ministry of Health guidelines that have information of their distribution by municipalities available [31, 32]. Most of the species were included by manual data processing based on the maps published by the Ministry of Health [32], while two species were from IUCN [33]. The list of species, maps of presence of each of the four genera, and their overlap are included in S2 Text. As the venomous snake richness variable was a new variable, we explored whether overlapping by species or genera would be best and decided on overlapping of genera, because data on species presence by municipality was incomplete in open access information available.

Demographic data on the socioeconomic variables were gathered from the Brazilian Institute of Geography and Statistics (acronym in Portuguese IBGE) [34, 35]. For the application of the decision-making tool to Rio Grande do Sul state, data provided by the State Health Department on the number of deaths by snakebite by municipality were included.

### Data analysis

In the descriptive analysis of the epidemiological situation, cases of snakebite and rates per 100,000 population were described by state and municipality using the major habitat type as background. In the table by state, the number of deaths and the fatality rate were included using data from the Ministry of Health webpage [12].

The spatial analysis consisted in computing zonal statistics and surface for the environmental variables, in addition to geocoding of the health outcome and socioeconomic data. ArcGIS and R zonal statistics by municipality (minimum, mean, maximum, standard deviation, range) were calculated for the elevation, temperature, and precipitation variables using a ‘raster’ package. Geo-processing geometric intersection of environmental features shaped the municipal surface of major habitat types. Natural breaks thematic mapping was conducted once the municipal statistics of the health outcome, environmental, and socioeconomic variables were geo-processed.

A spatial cluster analysis was done to identify concentrations of neighboring municipalities with a high number of snakebites. For the cluster analysis, Anselin Local Moran’s I method [36] was used to measure the spatial autocorrelation of snakebite among neighboring municipalities sharing a boundary or a vertex. The number of permutations was increased to get a p-value < 0.0001. Clusters of municipalities with high number of cases with positive autocorrelation are presented in the map, as well as clusters of low number of cases.

For the statistical analysis to measure the association of snakebite with possible drivers, first a univariate analysis was run with 12 variables being explored, and nine were preselected based on relative risk (RR) and 95% confidence interval (CI) of the estimates. Variance inflation factors (VIF) were estimated to verify the relations among all selected independent variables and to check for potential collinearity. None of the nine selected variables presented VIF greater than five and all stayed in the analysis. Also, Pearson correlation was performed on one-to-one variables and variables which showed correlation coefficient higher than 0.8 were excluded in the analysis.

The dependent variable was case count of snakebite per municipality and the independent variables were all nine possible drivers. For the regression models, the major habitat type variable was created as dichotomic (tropical: yes or no). As a limited (221/5,564) number of municipalities with zero cases was present in the database, the zero inflated models were not selected as final models. Poisson regression and negative binomial regression (NB) model were compared. We used deviance information criterion (DIC) performance as a goodness of fit test for the overall models. The model with lower DIC indicates better fit with data. Thematic mapping of snakebites over selected significant drivers was done.

### Instrument to support decision-making in risk analysis of snakebite

The flowchart to support decision-makers in the definition of higher risk areas to prioritize interventions was developed taking as reference a previous instrument created to categorize rabies risk in Brazil in the 1990s, and used by decision-makers for many years [37]. The instrument was developed to support state-level decision-makers, because in Brazil antivenom is distributed to municipalities from the state level.

**Fig 1:**
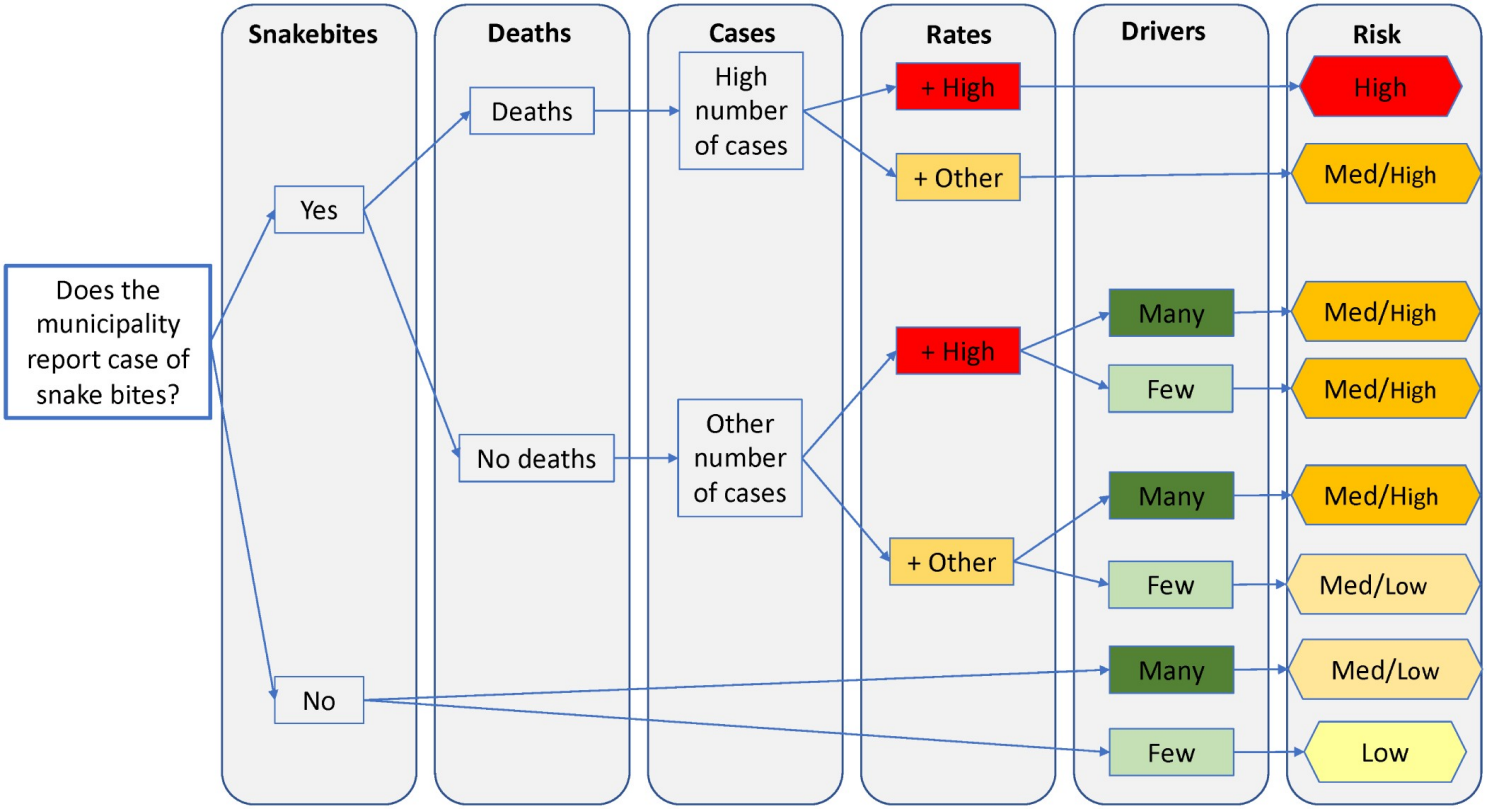
Flowchart to support decision-makers on snakebite risk by municipality in Brazil.

To define the cutoff ranges for the level of risk in the epidemiological situation, we used quartiles only with the state-level information (number of snakebites, rates, and occurrence of any deaths in the municipality). For the drivers selected in the final model, we defined the cutoff values of the quartiles (ranges) in the data for the entire country. Looking at the state-level data set, we identified higher risk quartiles by municipality, and added the number of drivers in the higher quartile of risk in that municipality. This part was done manually using Microsoft Excel. Each municipality was graded for level of risk of snakebite (High, Medium High, Medium Low, Low) and included in the flowchart (Fig 1).

## Results

### Descriptive and cluster analysis

**Fig 2:**
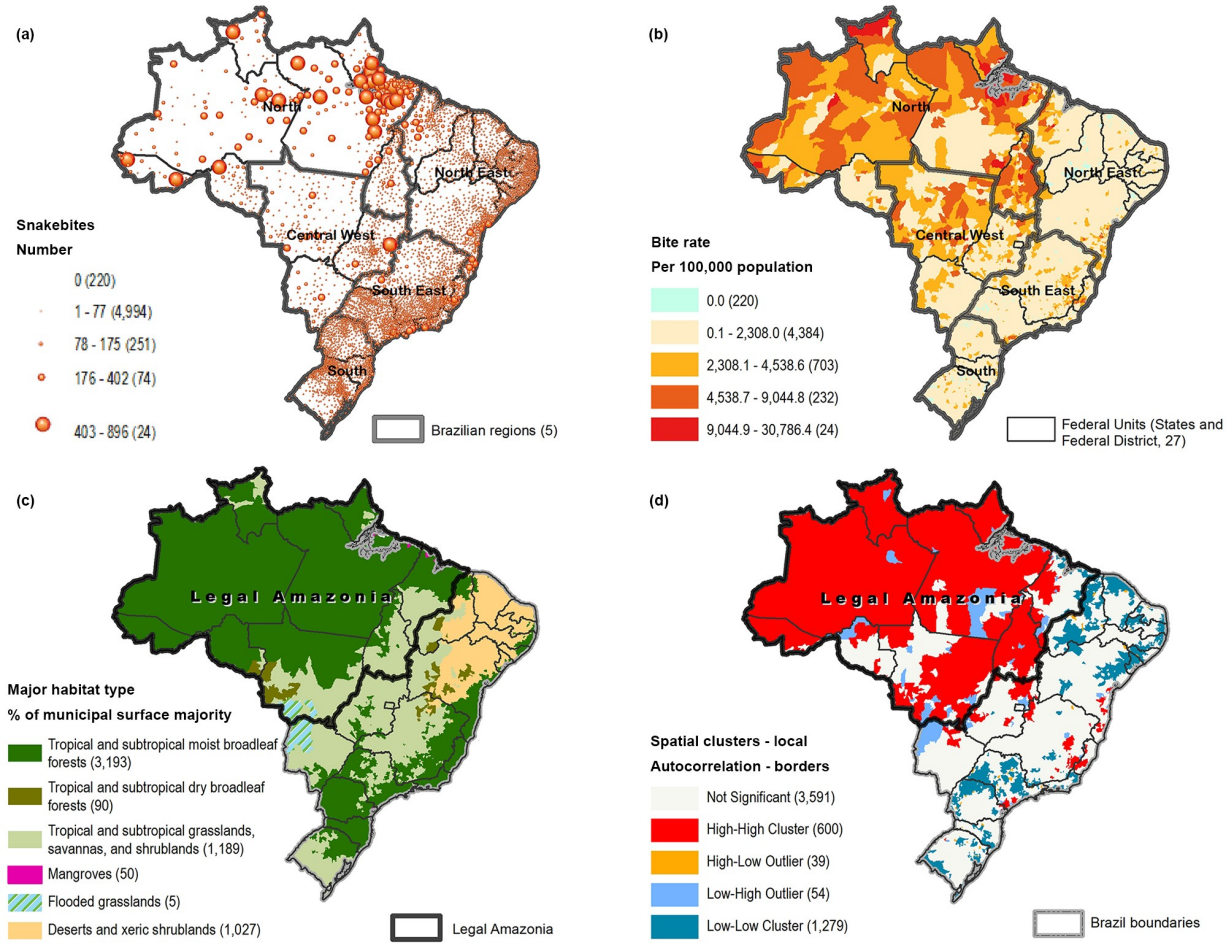
Number of snakebites (a), rates by 100,000 population (b), rates by municipality, major habitat type (c), and clusters of snakebites (d), Brazil, 2013–2017.

Between 2013 and 2017, 135,598 cases of snakebite envenoming were reported to SINAN, with an average of 27,120 cases per year at the country level. The country cumulative incidence rate in the period was 13.31 per 100,000 population. The higher number of snakebites and rates are in the North Region, and the lower number and rates are in the South Region (Table 1, Fig 2).

**Table 1:** Number of snakebite envenoming in the period, average cases per year, rate per 100,000 population, number of deaths, average deaths per year, and fatality rate, Brazil, by state and region, 2013–2017.

State	Pop. 2015	No. snakebites	Avg. cases	Rate per 100,000	No. deaths	Avg. deaths	% deaths
Rondônia	1,768,204	2,471	494	27.94	8	2	0.32
Acre	803,513	2,389	478	59.61	7	1	0.29
Amazonas	3,938,336	8,035	1,607	40.80	50	10	0.62
Roraima	505,665	1,842	368	77.13	14	3	0.76
Pará	8,175,113	25,210	5,042	61.67	82	16	0.33
Amapá	766,679	1,842	368	48.26	6	1	0.33
Tocantins	1,515,126	3,796	759	50.16	13	3	0.34
*North region*	*17,472,636*	*45,492*	*9,098*	*52.36*	*180*	*36*	*0.40*
Maranhão	6,904,241	6,991	1,398	20.25	43	9	0.62
Piauí	3,204,028	1,102	220	6.90	6	1	0.54
Ceará	8,904,459	3,084	617	6.93	15	3	0.49
Rio Grande do Norte	3,442,175	1,667	333	9.99	3	1	0.18
Paraíba	3,972,202	1,747	349	8.81	5	1	0.29
Pernambuco	9,345,173	3,894	779	8.49	22	4	0.56
Alagoas	3,340,932	1,607	321	9.64	1	0	0.01
Sergipe	2,242,937	788	158	7.04	7	1	0.89
Bahia	15,203,934	12,377	2,475	16.29	62	12	0.50
*Northeast region*	*56,560,081*	*33,257*	*6,651*	*11.82*	*164*	*33*	*0.49*
Minas Gerais	20,869,101	14,647	2,929	14.05	35	7	0.24
Espírito Santo	3,929,911	3,688	738	18.73	7	1	0.19
Rio de Janeiro	16,550,024	2,753	551	3.34	6	1	0.22
São Paulo	44,396,484	9,441	1,888	4.27	27	5	0.29
*Southeast region*	*85,745,520*	*30,529*	*6,106*	*7.13*	*75*	*15*	*0.25*
Paraná	11,163,018	4,167	833	7.52	13	3	0.31
Santa Catarina	6,819,190	3,515	703	10.32	8	2	0.23
Rio Grande do Sul	11,247,972	4,227	845	7.51	12	2	0.28
*South region*	*29,230,180*	*11,909*	*2,382*	*8.17*	*33*	*7*	*0.28*
Mato Grosso do Sul	2,651,235	2,570	514	19.61	6	1	0.23
Mato Grosso	3,265,486	6,010	1,202	36.81	28	6	0.47
Goiás	6,610,681	5,329	1,066	16.16	26	5	0.49
Distrito Federal	2,914,830	502	100	3.43	2	0	0.40
*Central-West region*	*15,442,232*	*14,411*	*2,882*	*18.71*	*62*	*12*	*0.43*
**Total**	**204,450,649**	**135,598**	**27,120**	**13.31**	**514**	**103**	**0.38**

Clusters of high number of snakebites municipalities are found mostly in the North Region and part of the Central-West Region that is considered the Legal Amazon Region of Brazil (Fig 2). Note that there are some low value clusters within areas of high number of snakebites. Considering the major habitat types, the analysis shows that clusters with high rates of snakebite are more predominant in the TSMBF major habitat (Fig 2). This biome represented 67.96% of snakebite cases in Brazil, with a total of 92,146 cases. Adding the other two types of tropical and subtropical major habitat (TS grasslands, savannas, and shrublands; TS dry broadleaf forests) amounts to 88.15% of the total cases in Brazil. Clusters of low-low values were most predominant in the Northeast Region in the major habitat type Deserts and Xeric Shrublands (DXS), and in parts of the Southeast Region (Fig 2).

Among the states, the average number of reported snakebite cases for the period ranged from 5,042 cases per year in the state of Para (population of 8,175,113) in the TSMBF major habitat, to 34 cases a year in the state of Rio Grande do Norte (population of 3,442,175) in the DXS major habitat.

The total number of deaths in the period was 514 (range by state 1–82), with an average per year of 103 (Table 1). The average fatality rate was 0.38 (range 0.01–0.89). Higher fatality rates were found in the states of the Legal Amazon Region and in the Northeast Region (Table 1).

At the second subnational administrative level, only 221 of 5,564 municipalities did not report any case of snakebite in the period; the maximum number of 896 snakebites during the five-year period was reported in one municipality in the North Region. The average number of bites in the period was 24.37 per municipality. In terms of their geographic distribution, 600 municipalities are significantly clustered in the Amazonia region with a high number of snakebites. A few are found along the length of the tropical forest known as Mata Atlantica.

**Fig 3:**
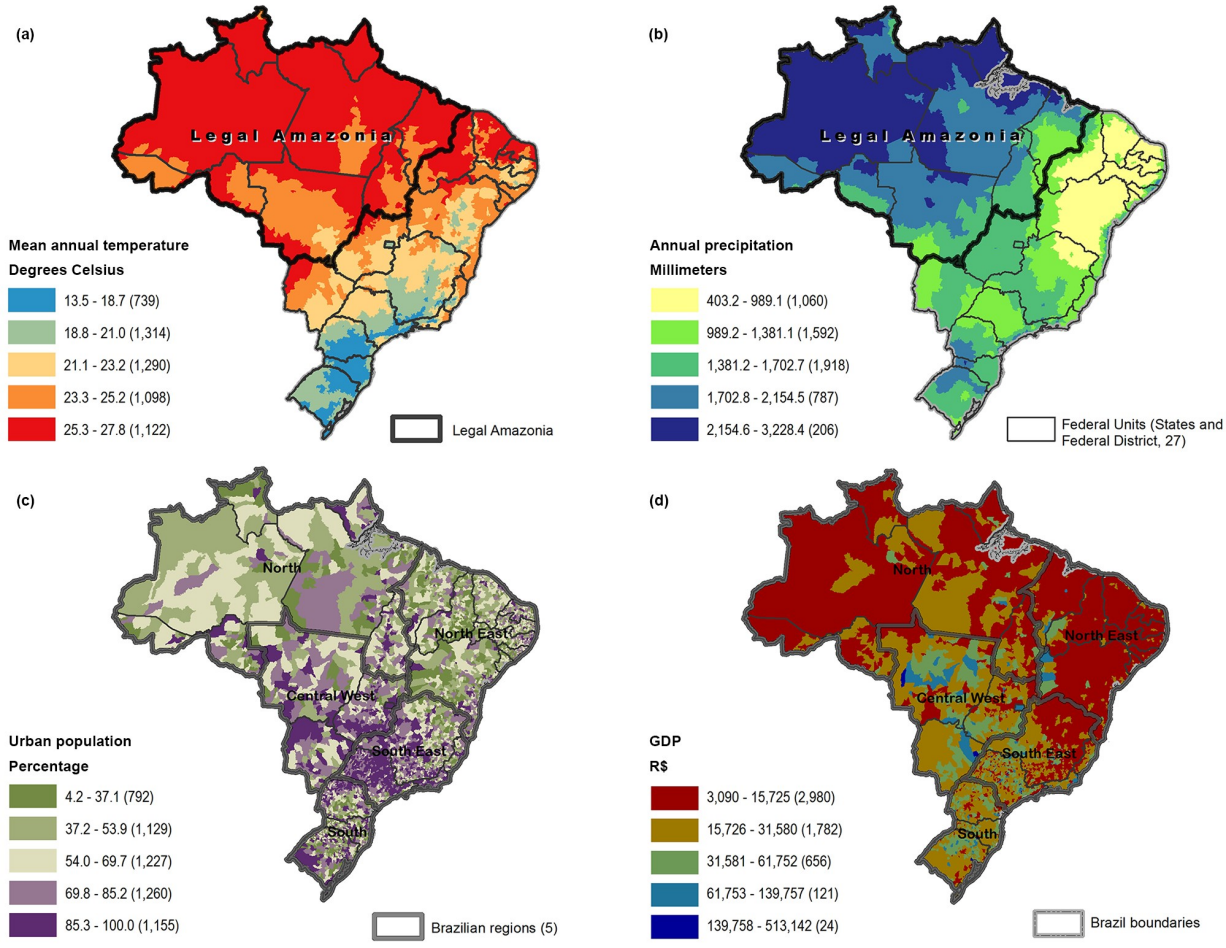
Spatial distribution of temperature (a), precipitation (b), percentage of urbanization (c), GDP per capita (d); by municipality, Brazil. Numbers in parentheses indicate municipalities in category.

For all variables, the mean, standard deviation, minimum and maximum values, and quartiles were obtained and described in Table A in S3 Text. Higher temperatures were found in the North Region in the TSMBF major habitat and in the Northeast Region, and lower temperatures in the South Region (Fig 3). Higher percentages of urbanization were in the Southeast and South regions (Fig 3), with similar distribution for the GDP per capita. Higher rates of precipitation were in the North Region in the TSMBF major habitat and in the Southeast Region in tropical and subtropical biomes, and lower rates in the Northeast Region with DXS major habitat. The average elevation in Brazil is 337 meters, as most of the territory is not high; however, the lower elevation areas in Brazil are found mostly in the North and in the South regions. Comparing the median rates of high and low incidence by the explanatory variables and the t-test p-value (Table B in S3 Text), it could be suggested that most of the variables presented significant differences between the two groups; only elevation did not present a difference.

**Fig 4:**
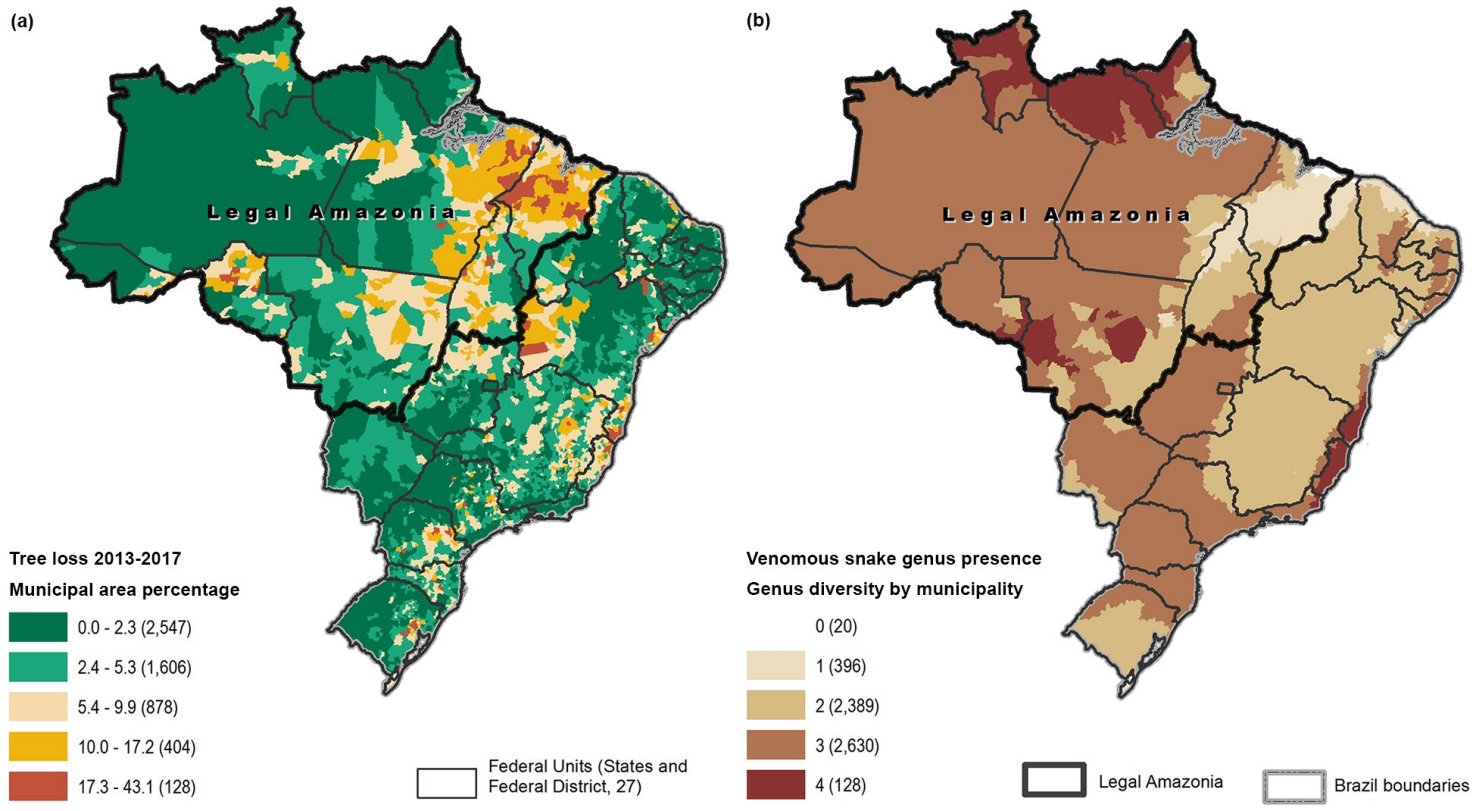
Spatial distribution of forest loss (a), and venomous snake genera (b) by municipality, Brazil. Numbers in parentheses indicate municipalities in category.

The forest loss variable presented many municipalities with higher rates of tree loss in the North Region, or Legal Amazon Region, where also most of the snakebites were reported (Fig 4). Maps showing the presence of each of the four genera and their overlap are included in S2 Text. The genus *Bothrops* appears to be present in almost all the territory; *Crotalus* outside the Amazon or far north; *Lachesis* mostly in the Amazon; and *Micrurus* in the Amazon and many other areas. The venomous snake richness variable presented higher overlapping in the Amazon Legal Region and in some coastal areas (Fig 4).

### Multivariate and drivers analysis

In the univariate analysis, none of the nine variables selected as independent covered the null value of the RR estimations with 95% confidence interval (CI) (Table E in S3 Text), and all variables were included in the full model. According to VIF estimated for all variables together and by one-to-one variable correlation, these nine variables did not present collinearity.

In the multivariate full model, eight of the variables considered as possible drivers did not cover the null value of the RR estimations with 95% CI (Table F in S3 Text): type of major habitat tropical or non-tropical (RR = 1.925; CI_95%_ = 1.755–2.11); temperature (RR = 1.647; CI_95%_ = 1.555–1.744); percentage of urbanization (RR = 0.507; CI_95%_ = 0.486–0.53); venomous snake richness (RR = 1.177; CI_95%_ = 1.132–1.224); precipitation (RR = 1.229; CI_95%_ = 1.183–1.277); elevation (RR = 1.235; CI_95%_ = 1.176–1.297); a weaker relation with forest loss (RR = 1.066; CI_95%_ = 1.04–1.093); and GDP per capita (RR = 0.957; CI_95%_ = 0.939–0.975). The variable forest cover (RR = 1.003; CI_95%_ = 1.002–1.004) was almost a borderline relation with the outcome variable; however, we do not consider this variable in the risk instrument analyzed below. The deviance performance was 41,232.2. Although other models were performed (Table F in S3 Text), this one was the best fit.

### Application of the instrument to the state of Rio Grande do Sul

This instrument was created to support decision-makers at the state level; however, the range of drivers was created based on the variables for the whole country. The higher quartiles for each variable are described in Table A in S4 Text. The presence of four or more drivers in a municipality was considered as “many” drivers. The flowchart was applied to Rio Grande do Sul state as an example (Fig A in S4 Text). The exercise was performed using a spreadsheet with all information related to this state from the same period (2013–2017). The epidemiological situation of the state and whether the municipality had many drivers is presented below and in the flowchart in Fig A in S4 Text. The number of cases of snakebite in the period was 4,227

**Fig 5:**
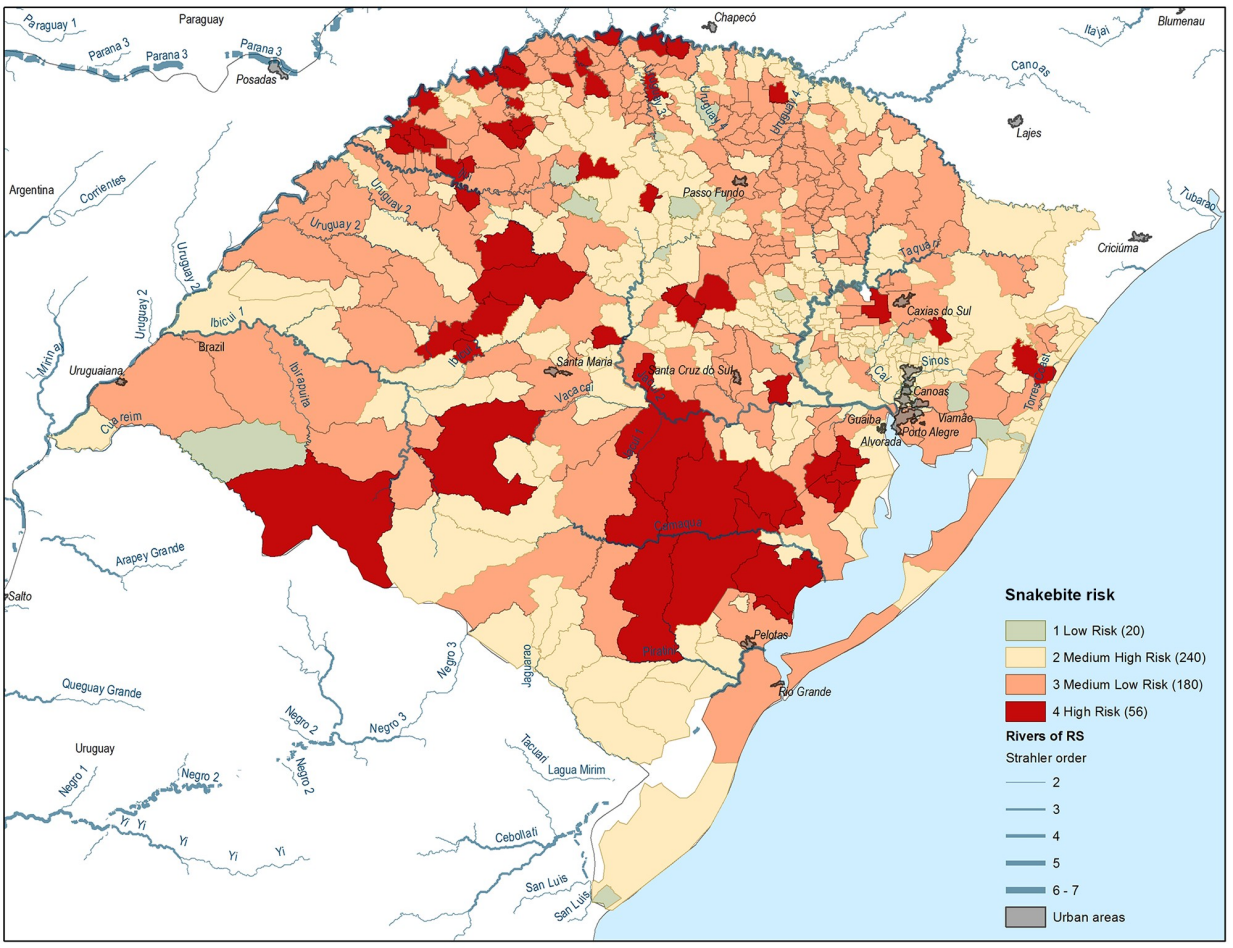
Stratification for risk of snakebite, Rio Grande do Sul state, Brazil, 2013–2017.

- Number of municipalities with snakebite cases: 462/496
- Number of municipalities with deaths by snakebite: 10/496
- Number of municipalities with “high risk”: 56
- Number of municipalities with “medium high risk”: 217
- Number of municipalities with “medium low risk”: 203
- Number of municipalities with “low risk”: 20
- Most of the municipalities of Rio Grande do Sul were classified as medium low risk and medium high risk according to this instrument (Fig 5 and Fig A in S4 Text).

## Discussion

Snakebite cases are distributed across the entire country, with only 0.5% of municipalities not reporting on cases during the study period. This is to be expected due to Brazil’s environmental characteristics, among which is that more than half of the territory has a TSMBF major habitat type. As around 27,000 cases are reported annually to SINAN, snakebite envenoming is a major public health problem. All these cases need health care that includes clinical evaluation and treatment with antivenom free of charge.

Our analysis shows a particularly high concentration of snakebite cases in the Legal Amazon Region of Brazil (Fig 2). This region is extremely complex: an expansive floodplain, formed by its vast rivers, with huge biodiversity, although with ongoing deforestation and large impacts on its indigenous peoples and the natural world [38]. The North Region has lower GDP per capita and lower rates of urbanization than the rest of Brazil, and previous studies have demonstrated that snakebite is a poverty-related disease and a disease mainly affecting rural populations [1, 39]. Our study supports this observation, suggesting protective association of urbanization with snakebites and a borderline association with lower GDP.

Some environmental drivers, such as temperature and precipitation, have been associated with snakebite in previous studies [17, 40]. There are some descriptive studies pointing out that some of the victims of snakebite accidents in the state of the Amazon were local loggers or others who lived off the extraction of products from the forest [4, 5]; however, the association of snakebite with forest loss in Brazil is a new finding. Our study supports this observation on a broader scale. Environmental preservation and agricultural production policies are a major concern globally, especially when these involve the Amazonian region [41, 42]. Taking into consideration the complexity of this topic, our findings suggest that the largest areas with concentration of snakebite over forest loss (Fig 4) are located in the eastern part of Para state (Amazon Region), where significant areas were designated for agrarian settlements [43]. The municipality with the highest number of snakebites in the study is Santarem (896 cases in the study period), which is located at the confluence of the Amazon and Tapajos rivers. Santarem city is one of the oldest in the state and was home to an advanced indigenous population in pre-colonial times. In this region, areas such as the Tapajós National Forest, the Extractive Reserve and the Amazon River floodplain are examples of environmental diversity [44]. However, in recent decades, this natural landscape has been significantly altered with the emergence of pastures for livestock in the early 1970s, and more recently, the replacement of these pastures by soybean plantations that interfere with the structure of snake communities, especially for *Bothrops atrox* [44]. *Bothrops atrox* is a very abundant species in the Santarem area and in the entire Amazon Region, and is considered one of the most dangerous snakes in South America, known for its defensive behavior and frequent involvement in snakebite accidents [44, 45].

The other municipality with a high number of cases in the same area is Breves (861 cases) on Marajo island, the largest river island in Brazil, with an economy based on agriculture and water buffaloes [46]. On Marajo island, two main ecosystems are described: natural grasslands (open area) and forest. Most of the open area remains flooded during several months of the year due to rainfall. A study on the taxocenose of snakes on Marajo island considers the island unique in terms of its snake species composition and calls for these distinctive ecosystems to be preserved [47].

Transdisciplinary studies with a One Health vision are needed to better understand the relationships in the animal–human–ecosystem interface in this vast territory that makes up the Legal Amazon Region of Brazil, and how economic interests are affecting the number of snakebite accidents.

In the cluster analysis there is a visible concentration of municipalities with high numbers of snakebites in the Legal Amazon Region of Brazil (Fig 2). However, there are some areas with low numbers of cases amid areas with high incidences of snakebites. Studies are needed on whether this population has access to hospitals with antivenom. It may be that some of these areas are indigenous reservations; for example, one area in the south of Para state is similar to the mapped area of officially defined indigenous land [43].

Only little information is available about snakebites in the Brazilian indigenous population, and underreporting of cases is likely, due to limited access to health care in remote areas of the Amazon, where travel involves covering large distances by boat [48, 49]. It is observed that late care is the main risk factor for a poor clinical prognosis. Local and systemic complications are factors that are directly associated with the opportunity of treatment and availability of antivenom. Indigenous populations in the Amazon Region are at high risk for snakebites due to the environmental conditions, combined with a scenario of high social vulnerability. They may experience more difficulty accessing health care with the antivenom available, since they live in more remote areas and the antivenom needs to be refrigerated for storage. The Ministry of Health of Brazil has identified the need to strengthen the availability of antivenom in the indigenous territories and has initiated a pilot project to provide antivenom to these areas where there is cold chain infrastructure [50]. Also, trainings on snakebite treatment for health professionals working in indigenous territories are being developed.

According to a government document [43], 40% to 70% of the territory of the states of the North Region of Brazil is legally protected as conservation zones and indigenous land. However, this does not necessarily mean that this protection is being enforced, and illegal prospecting, land grabbing, and deforestation are frequently reported in the Legal Amazon Region [51–53].

According to a publication by the Ministry of Health of Brazil, accidents with snakes are the most frequent accidents involving venomous animals in Brazil (out of the 9,406 work-related accidents with venomous animals reported from 2007 to 2017, 4,282 cases were snakebites), mostly among males of productive age and in rural areas [10]. The use of personal protective equipment should therefore be recommended to all high-risk occupational groups. One of the recommendations of the Ministry of Health publication was to organize training for the medical teams in these areas, which is already happening [54, 55].

The variable related to the overlapping of venomous snake genera, higher in the Legal Amazon Region, stayed in the final model, suggesting an association with snakebite risk. This finding was expected, given the richness of venomous snakes in this area [5, 45, 47]. Our maps presented similar areas of higher risk for snakebite as the publication of Longbottom et al. [17], with concentration of cases in the Amazon Region and in the Atlantic Forest on the southeast coast.

One of the possible limitations of this study is the use of aggregated data by municipality, as ecological studies are commonly associated with ecological fallacy [56]. However, our geographical approach helped to gather, unify, and shape all factors (environmental, socioeconomic, and health outcome) into the same area and to detect spatial patterns. Previous studies used this methodology to identify risk areas for snakebites in other countries [18, 19]. The methodology presented in this study aims to support decision-makers in the identification of higher risk municipalities for snakebite interventions; more disaggregated studies are suggested to evaluate individual risk factors for snakebites.

The state of Rio Grande do Sul is dedicated mostly to agrobusiness, and previous studies bring to attention that certain characteristics of agroecosystems are key determinants of the human–livestock– snake conflict, and that some agricultural practices can intensify snakebite risk [60]. We recommend more studies among the rural population of this state and others, using the One health approach. In the Rio Grande do Sul example of risk stratification, the majority of municipalities were classified as medium risk, which coincided with the level of risk of the state in relation to other states of Brazil. However, since most of the municipalities presented snakebites during the study period, antivenom needs to be available state-wide. There were several municipalities of higher risk around the Camaca river and tributaries, where rice and tobacco plantations are an important part of the productive processes of this region [22]. Previous studies in Asia demonstrated the association between snakebites and rice workers, and snakebite is one of the risks identified for tobacco workers in different parts of the world, mostly for illegal child laborers [57–59].

With a total area of 8,515,767.049 km2 and more than 5,500 municipalities, for Brazil this not only involves a very large amount of antivenom (in 2017, 234,194 ampoules were distributed to 2,212 hospitals free of charge), but the fact that the majority of cases are in areas with forest major habitat type with lower GDP and a lower percentage of urbanization, creates additional challenges for service delivery. The distribution and maintenance of the antivenom supply in remote areas is crucial. In the Legal Amazon Region, transportation to larger cities often takes several hours by boat, which increases the chances that a significant number of snakebite victims could have difficulty reaching a hospital with antivenom in time, leading to disability or death. Research into types of antivenom that are safe, effective, at reasonable cost, more easily administered, with longer shelf life, and without the need for refrigeration, would be especially useful. Strengthening the laboratory capacity of antivenom producers and research into new therapies, diagnostics, and medical interventions, are recommendations of several publications and part of the WHO strategy [3, 6, 14].

## Conclusion

This study brings evidence that saving the lives of snakebite victims in Brazil is a major activity of the health system, and the fact that the majority of cases are in areas with forest major habitat type with lower GDP and a lower percentage of urbanization, creates additional challenges for service delivery. The distribution and maintenance of the antivenom supply in remote areas is crucial. Research into types of antivenom with a longer shelf life without the need for refrigeration is needed. It is urgent to fight against this potentially life-threatening disease, which is preventable by making safe and effective antivenom more widely available and accessible, and by raising awareness on primary prevention among communities, health workers, and agricultural workers in high risk areas, particularly if they are rural and remote, such as the Amazon Region. We also recommend more in-depth research on the incidence of snakebites among Brazil’s indigenous population, and their access to health care services that provide lifesaving antivenom.

## Supporting information

S1 Text Variables included in the study. Selected variables and sources of information used to create a database by municipality.
(DOCX)

S2 Text Snake richness variable. Venomous snakes present in Brazil listed by World Health Organization (WHO), the Ministry of Health of Brazil, and the International Union for Conservation of Nature (IUCN).
(DOCX)

S3 Text Statistical analysis. Results of the descriptive, univariate, and multivariate analyses.
(DOCX)

S4 Text Flowchart risk instrument results. Results applied to Rio Grande do Sul state, flowchart to support decision-makers on snakebite risk by municipality in Brazil.
(DOCX)

S5 Text Alternative Language Abstract (Portuguese). (DOCX)

S6 Text Alternative Language Abstract (Spanish). (DOCX)

S7 Text STROBE Checklist. (DOC)
