# Supplementary material for: Overview of snakebite in Brazil: Possible drivers and a tool for risk mapping
Source: PLoS Negl Trop Dis. 2021 Jan 29;15(1):e0009044. doi: 10.1371/journal.pntd.0009044 (PMC7875335; doi:10.1371/journal.pntd.0009044)
Supplement: S1 Text — Selected variables and sources of information used to create a database by municipality. (DOCX) [file pntd.0009044.s001.docx]

**Supporting information 1. Variables included in the study**

**Table A**. Selected variables and sources of information used to create a database by municipality.

| **Category** | **Variables** | **Period** | **Units** | **Data sources** |
| --- | --- | --- | --- | --- |
| Outcome | Snakebite cases | 2013–2017 | (count) | SINAN ^1^ |
| Demographic | Total population | 2015 | (count) | IBGE ^2^ |
|  | % of urban population | 2015 | % | IBGE ^2^ |
| Environmental | Forest loss ^a^ | 2013–2017 | % | Global Forest Change ^3^ |
|  | Temperature | 1970–2000 | ^o^C | WorldClim ^4^ |
|  | Precipitation | 1970–2000 | Mm | WorldClim ^4^ |
|  | Elevation | - | M | SRTM v 4.1 ^5^ |
|  | Venomous snake genera richness ^b^ | - | (count) | IUCN ^6^, Ministry of Health of Brazil |
|  | Major habitat type ^c^ | - |  | FAO ^7 8 9^ |
|  | Tree cover ^a^ | 2000 | km^2^ | \| Global Forest Change ^3^ \| \| --- \| |
| Socioeconomic | GDP per capita | 2016 | Reais | IBGE ^10^ |

Sources:

^1^ SINAN - Acidentes por animais peçonhentos – Notificações registradas no sistema de informação de agravos de notificação [Internet]. Brasília, Brasil: Ministério da Saúde. c 2019 - [cited 2019 June 17]. Available from: <http://tabnet.datasus.gov.br/cgi/deftohtm.exe?sinannet/cnv/animaisbr.def>

^2^ IBGE - Fundação Instituto Brasileiro de Estatística. 2019. [cited 2019 August 18]. Brasilia, Brasil: Ministério da Saúde. c 2017. Available from: <http://downloads.ibge.gov.br/downloads_estatisticas.htm>.

<https://www.ibge.gov.br/estatisticas/sociais/populacao/9103-estimativas-de-populacao.html?edicao=17283&t=downloads> [cited 2019 October 09]

^3^ Hansen MC, Potapov PV, Moore R, Hancher M, Turubanova SA, Tyukavina A, et al. High-resolution global maps of 21st-century forest cover change. *Science* 2013;342:850–853. [cited 2019 August 18 ???]. Available from: <http://earthenginepartners.appspot.com/science-2013-global-forest>.

^4^ Fick SE, Hijmans RJ. Worldclim 2: New 1-km spatial resolution climate surfaces for global land areas. *International Journal of Climatology* 2017;37(12):4302-4315. [cited 2019 August 18 ???]. Available from: <http://worldclim.org/version2>

^5^ Jarvis A, Reuter HI, Nelson A, Guevara E. 2008. Hole-filled SRTM for the globe Version 4, available from the CGIAR-CSI SRTM 90m. [cited 2019 August 18 ???]. Available from: <http://srtm.csi.cgiar.org>.

^6^ IUCN 2019. The IUCN Red List of Threatened Species. 2019-2. [cited 2019 July 1]. Available from: https://www.iucnredlist.org.

^7^ FAO. FAO-GeoNetwork-geo-spatial - WWF Global Ecoregions Map [Internet]. [cited 2014 Dec 10]
<http://www.fao.org/geonetwork/srv/en/resources.get?id=1009&fname=1009.zip&access=private>

^8^ WWF Global - Major Habitat Types: <http://wwf.panda.org/about_our_earth/ecoregions/about/habitat_types/>

^9^ United Nations Environment Programme (UNEP) 2014 [http://ede.grid.unep.ch//mod_download/download_geospatial.php?selectedID=1814&newFile=download/wwf_ecoreg_tot_po_shp.zip](http://ede.grid.unep.ch/mod_download/download_geospatial.php?selectedID=1814&newFile=download/wwf_ecoreg_tot_po_shp.zip)

^10^ IBGE - Fundação Instituto Brasileiro de Estatística. 2019. [cited 2019 August 18]. Brasilia, Brasil: Ministério da Saúde. c 2017. Available from: <http://servicodados.ibge.gov.br/Download/Download.ashx?u=ftp.ibge.gov.br/Pib_Municipios/2016/base/base_de_dados_2010_2016_xls.zip>

**^a^ Forest loss and tree cover**

The authors obtained the variable as raster type data, which were composed of 30m pixels that contain values for both variables: forest cover and forest loss. The forest cover variable was provided for the year 2000 and ranged from 0% to 100%, indicating the probability of forest being present within each pixel. Pixels with ≥50% forest cover were considered to indicate forest presence in our study. Forest loss was provided as a categorical variable indicating which year there were tree-loss events between 2001 and 2017. We assessed the deforestation level in 2016 as proportion of pixels indicating tree-loss events in 2016 among pixels of forest presence for each municipality (Min 2019).

According to the authors (ref <https://earthenginepartners.appspot.com/science-2013-global-forest/download_v1.6.html>) tree cover is defined as canopy closure for all vegetation taller than 5m in height (encoded as a percentage per output grid cell, in the range 0–100t); and forest loss (tree loss) as a stand-replacement disturbance, or a change from a forest to non-forest state.

Data preprocessing for tree cover and tree loss was the same methodology used in previous publication (Min 2019, <https://www.mdpi.com/1660-4601/16/9/1518> ).

**^b^ Snake richness** in S2.

^c^ **Major habitat type**

According to FAO and WWF, eco-region units (867) are grouped into [14 major habitat types](https://www.worldwildlife.org/biome-categories/terrestrial-ecoregions) (MHT), which are defined as relatively large units of land or water containing a distinct assemblage of natural communities sharing a large majority of species, dynamics, climatic, and other environmental conditions.

**Table B.** Differences in the municipalities in previous (2008) and current (2018) shapefile by municipality, Brazil

| The identification number of municipalities that were disaggregated  (total number of municipalities = 5564) |  | The new identification number of municipalities  (total number of municipalities = 5570) |
| --- | --- | --- |
| 150680 | = | 150680 + 150475 |
| 221100 | = | 221100 + 220672 |
| 420700 | = | 420700 + 422000 |
| 420940 | = | 420940 + 421265 |
| 430210 | = | 430210 + 431454 |
| 500325 | = | 500325 + 500627 |
| 500295 | = | 500295 + 500627 |
| 500020 | = | 500020 + 500627 |
