# Supplementary material for: Overview of snakebite in Brazil: Possible drivers and a tool for risk mapping
Source: PLoS Negl Trop Dis. 2021 Jan 29;15(1):e0009044. doi: 10.1371/journal.pntd.0009044 (PMC7875335; doi:10.1371/journal.pntd.0009044)

**Supporting information 2. Snake richness variable**

**Table A**. Venomous snakes present in Brazil listed by WHO, the Ministry of Health of Brazil, and IUCN

| Genera | WHO list | Brazil Ministry of Health list | IUCN list | Included in the study and source |
| --- | --- | --- | --- | --- |
| *Bothrops* | *Bothrocophias hyoprora* | No |  |  |
|  | *Bothrocophias microphthalmus* | No |  |  |
|  | *Bothrops alternatus* | Yes |  | X ^1^ |
|  | *Bothrops atrox* | Yes |  | X ^1^ |
|  | *Bothrops bilineatus* | No |  |  |
|  | *Bothrops brazili* | No |  |  |
|  | *Bothrops diporus* | No |  |  |
|  | *Bothrops jararaca* | Yes |  | X ^1^ |
|  | *Bothrops jararacussu* | Yes | Yes | X ^2^ |
|  | *Bothrops leucurus* | No |  |  |
|  | *Bothrops mattogrossensis* | No |  |  |
|  | *Bothrops moojeni* | Yes |  | X ^1^ |
|  | *Bothrops neuwiedi* | Yes |  | X ^1^ |
|  | *Bothrops pubescens* | No |  |  |
|  | *Bothrops spp.* | No |  |  |
|  | *Bothrops taeniatus* | No |  |  |
|  | No | *Bothrops erythromelas* |  |  |
| *Crotalus* | *Crotalus durissus* | Yes | Yes | X ^2^ |
| *Lachesis* | *Lachesis muta* | Yes |  | X ^1^ |
| *Micrurus* | *Micrurus corallinus* | Yes |  | X ^1^ |
|  | *Micrurus lemniscatus* | Yes |  | X ^1^ |
|  | *Micrurus spixii* | No |  |  |
|  | *Micrurus spp.* | No |  |  |
|  | *Micrurus surinamensis* | No |  |  |
|  | No | *Micrurus frontalis* |  |  |

Sources: ^1^ Brazil Ministry of Health; ^2^ IUCN

**Figure A.** Maps of presence of venomous snakes in Brazil according the Ministry of Health of Brazil Guidelines ^1^ or IUCN ^2^ transformed by authors in municipalities included in the study

*Bothrops alternatus ^1^*


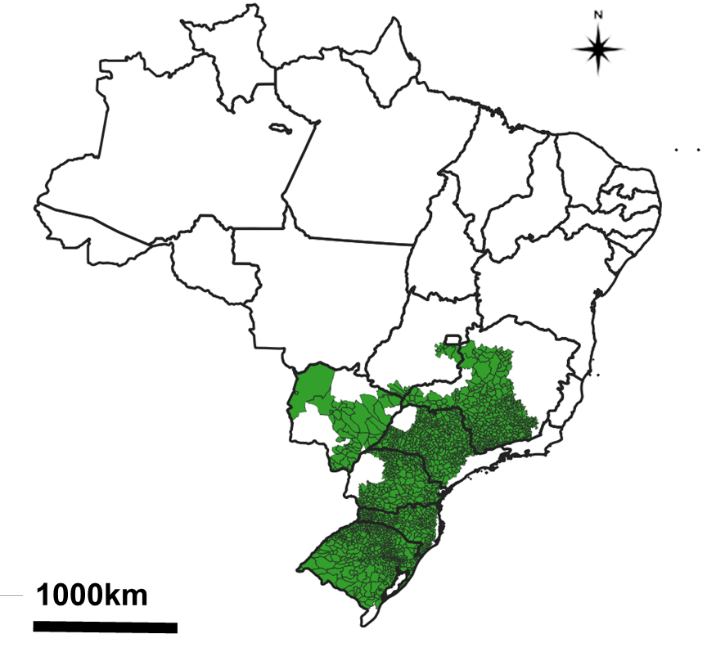


*Bothrops atrox ^1^*

*
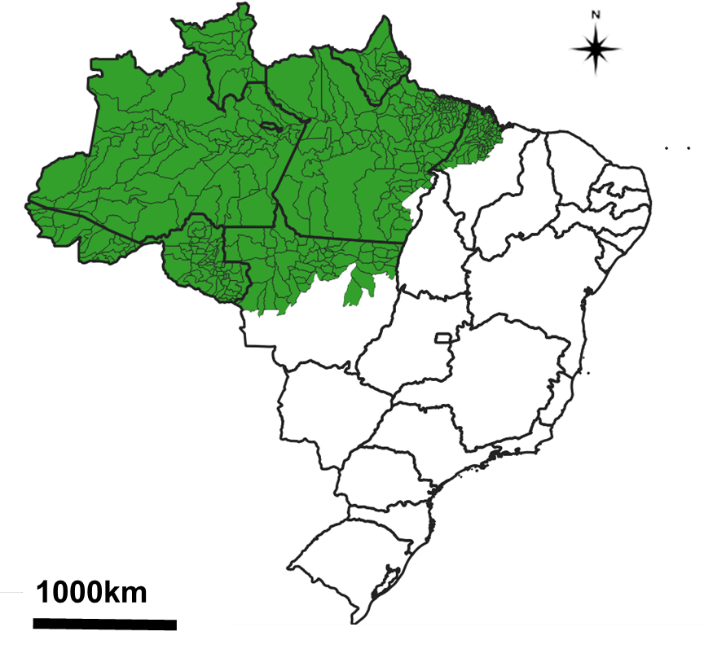
*

*Bothrops jararaca ^1^*


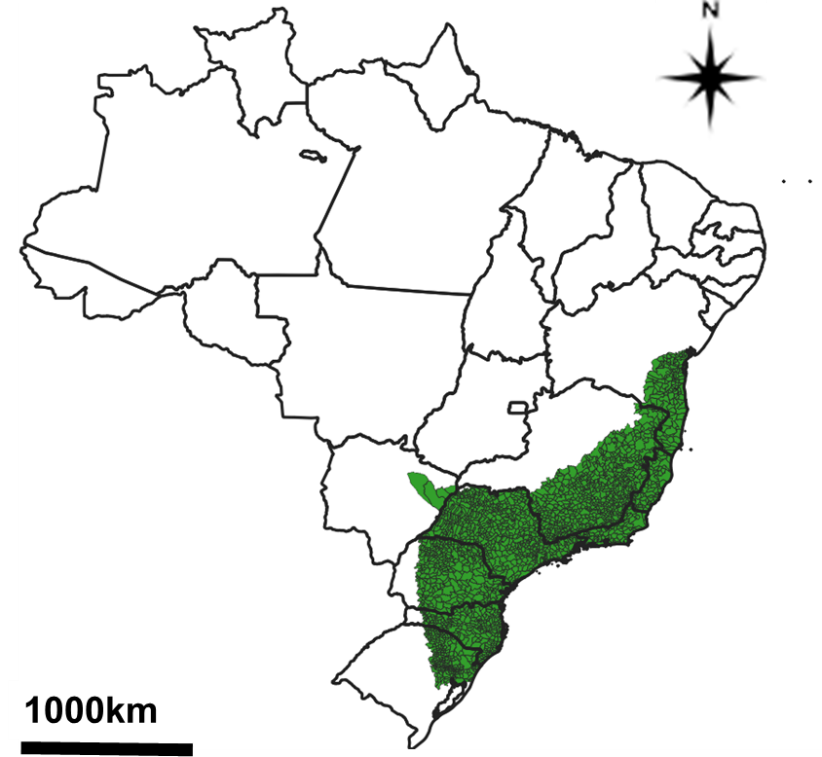


Bothrops jararacussu *^2^*


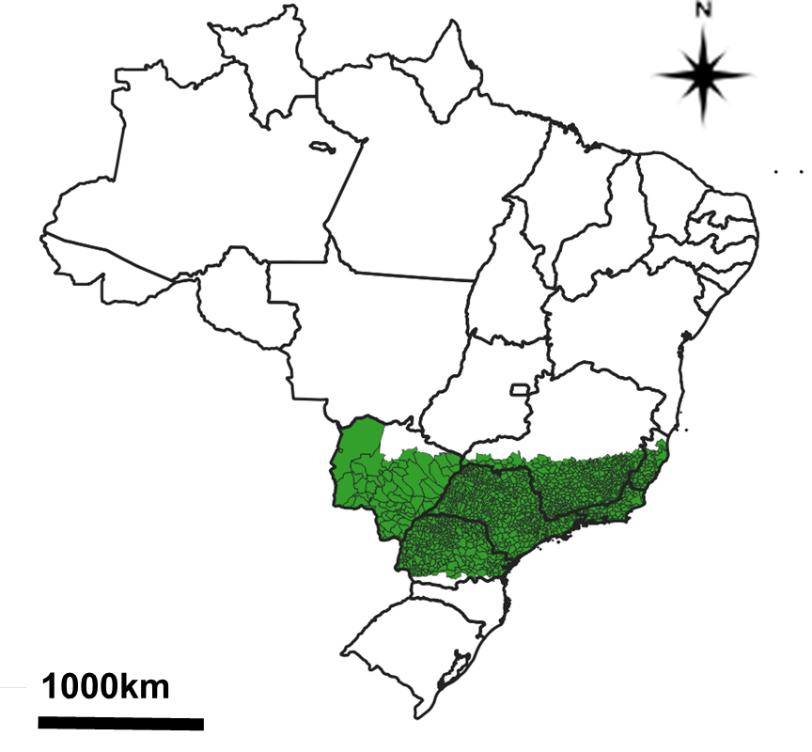


*Bothrops moojeni 1*


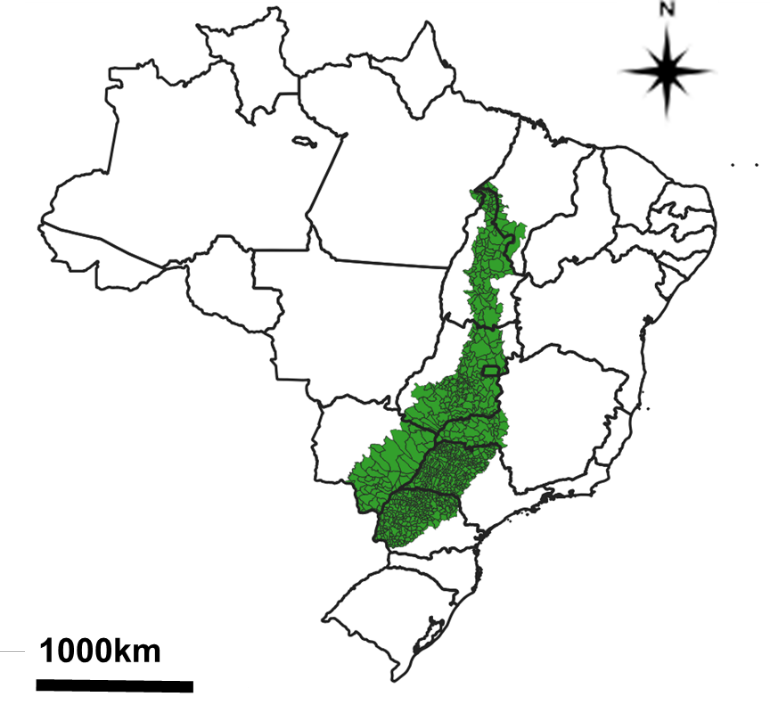


*Bothrops neuwiedi ^1^*


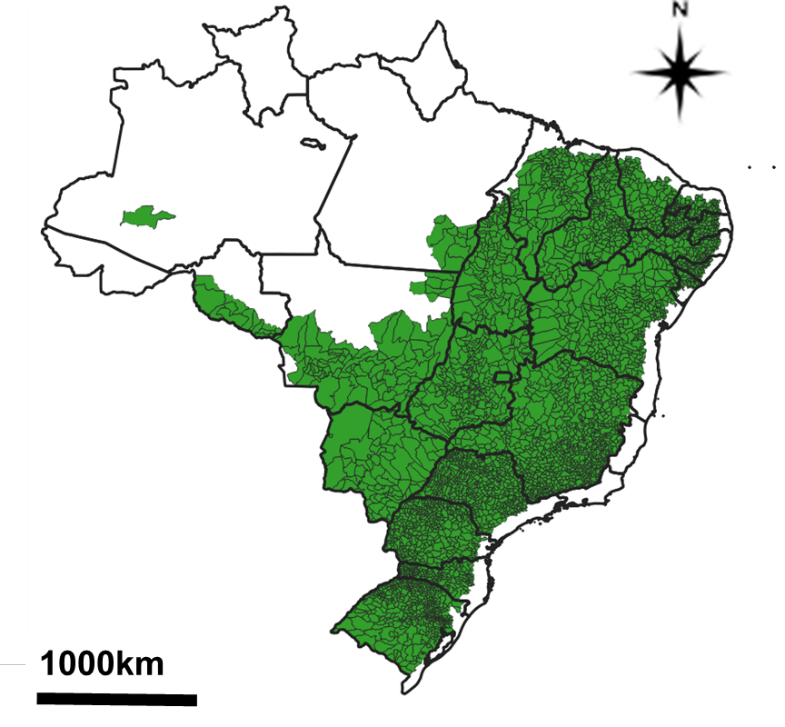


*Crotalus durissus ^2^*


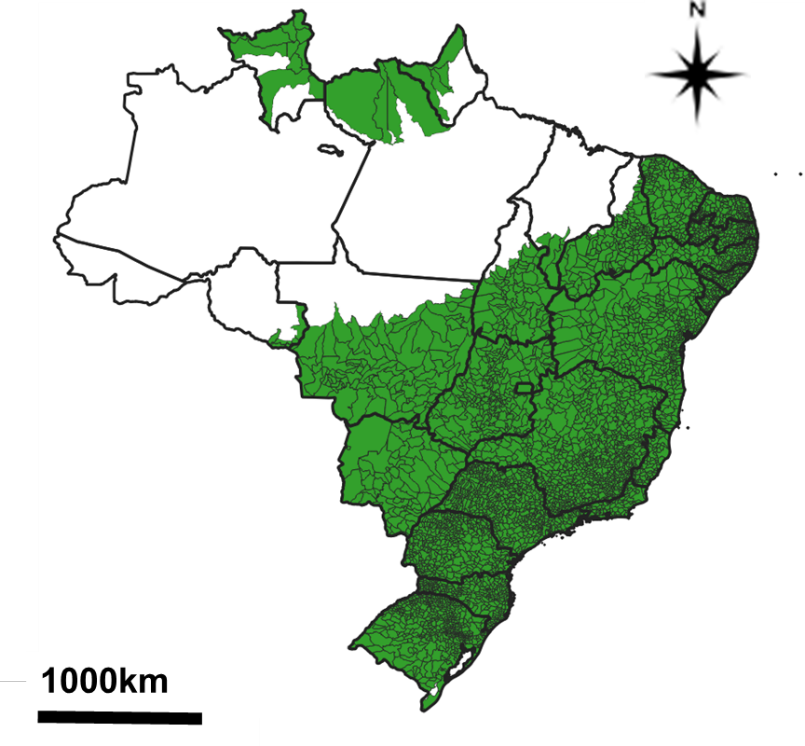


*Lachesis muta* ^1^


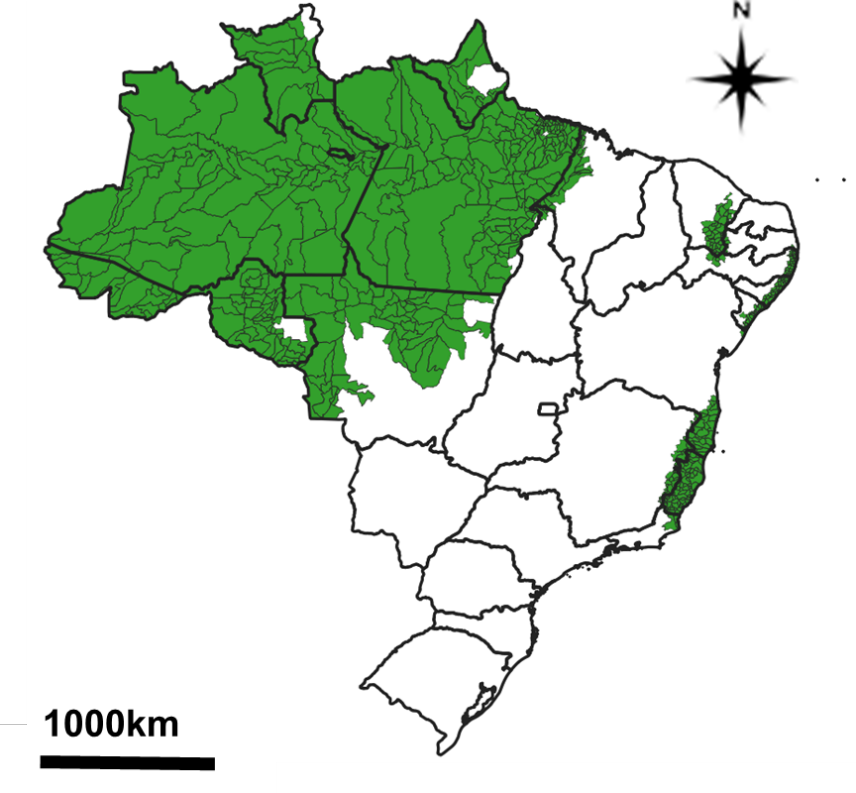


*Micrurus corallinus* ^1^


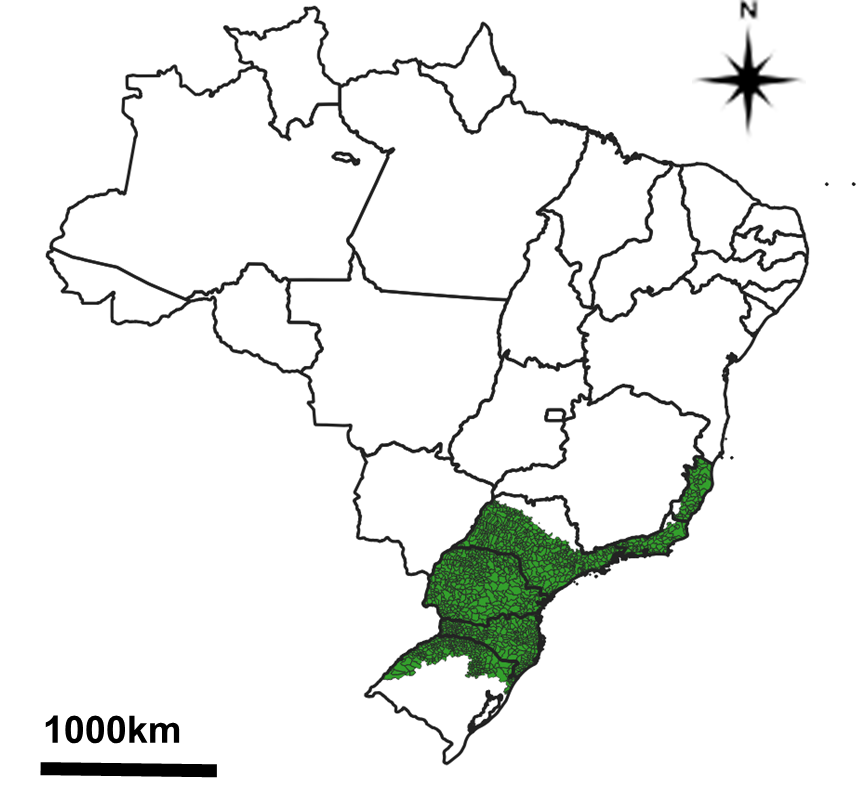


*Micrurus lemniscatus* ^1^


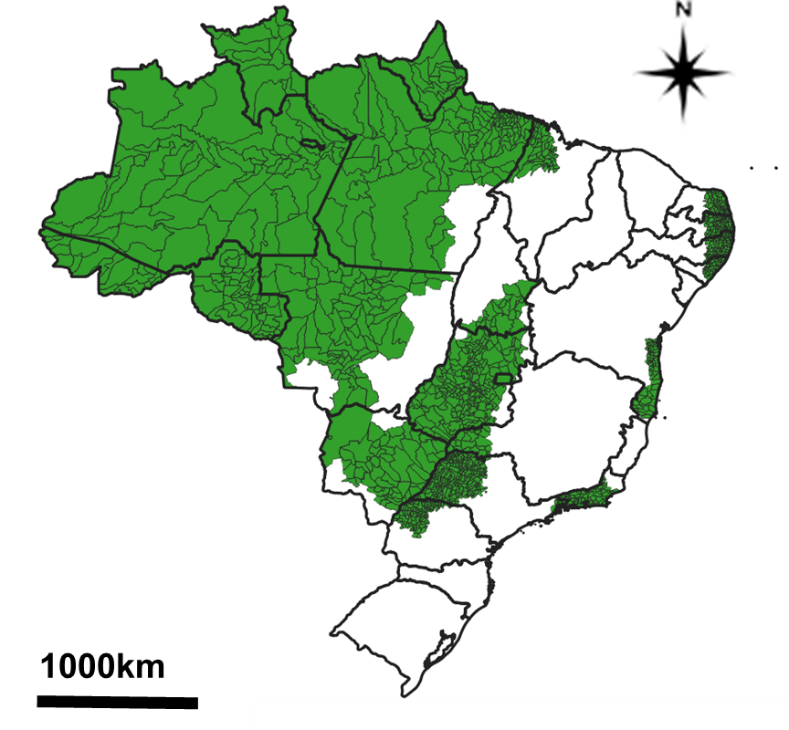


**Figure B.** Presence of the genus *Bothrops* by municipality in Brazil


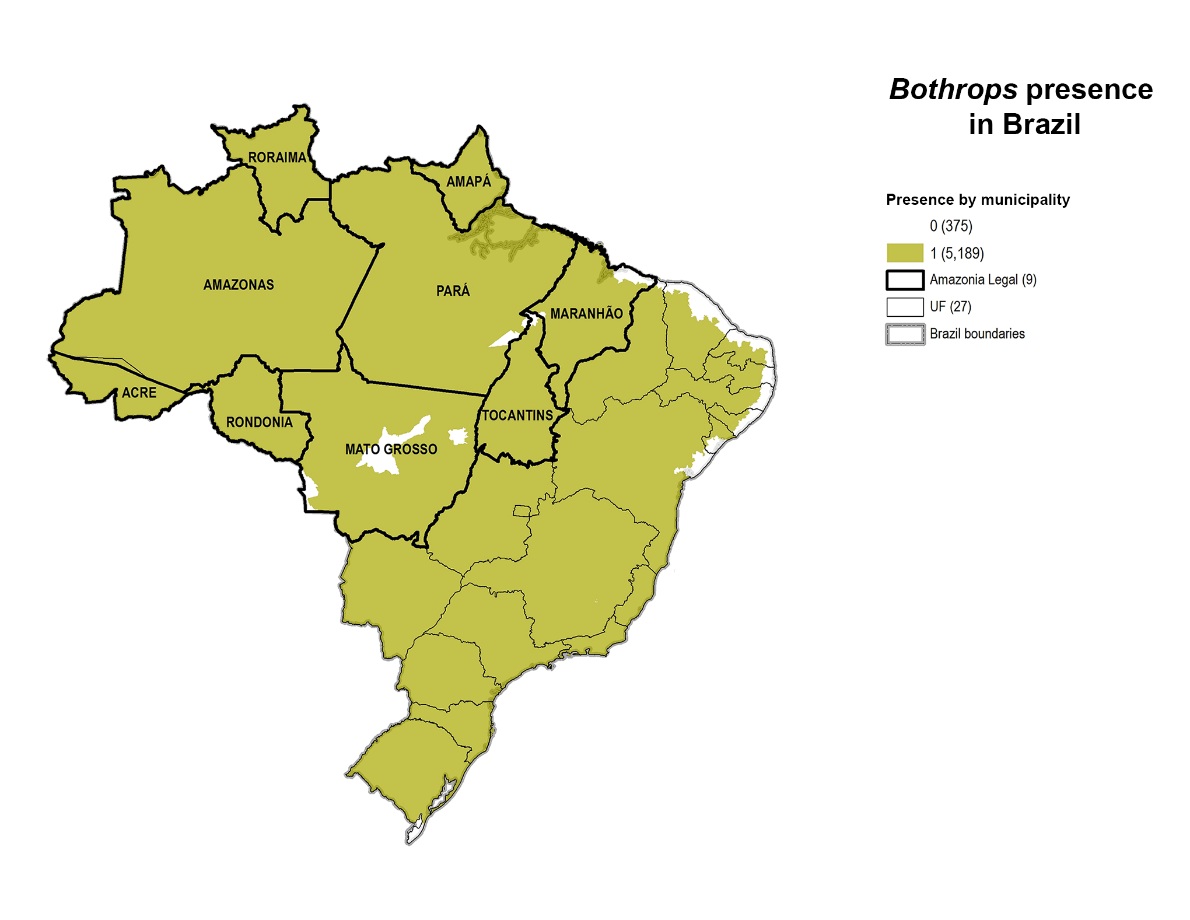


**Figure C.** Presence of the genus *Crotalus* by municipality in Brazil


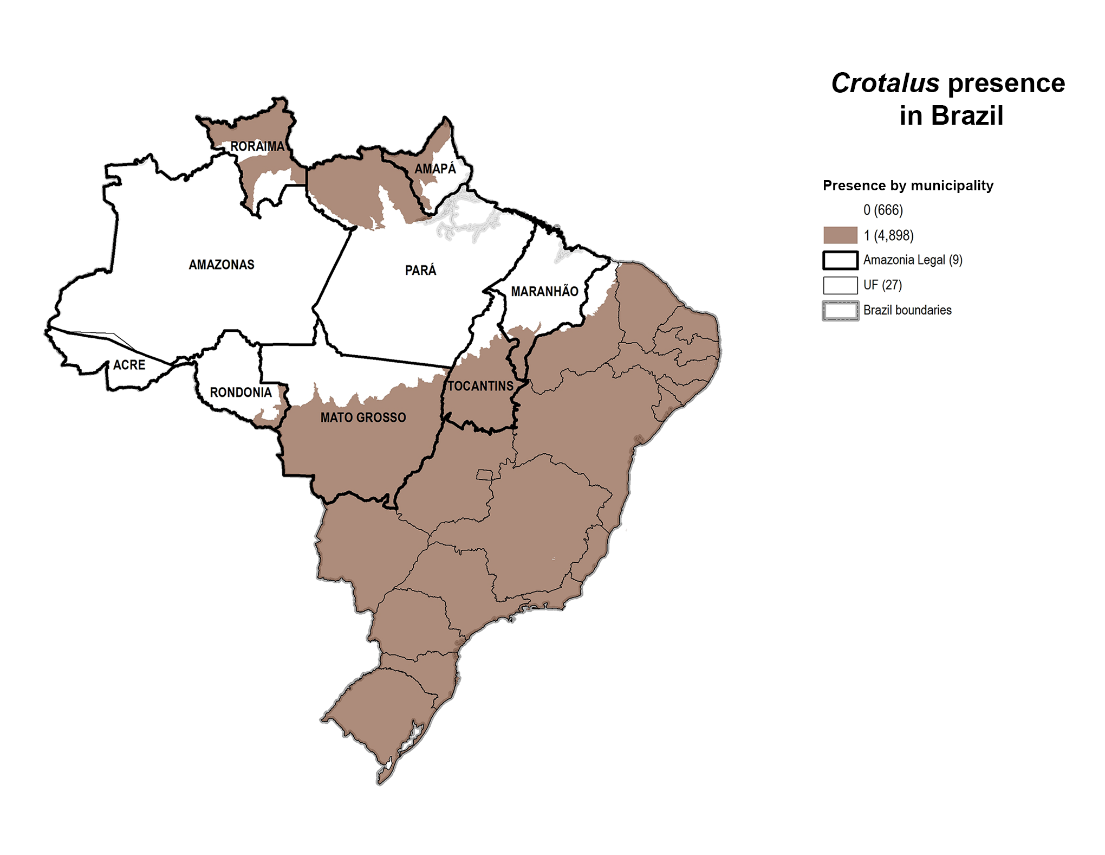


**Figure D.** Presence of the genus *Micrurus* by municipality in Brazil


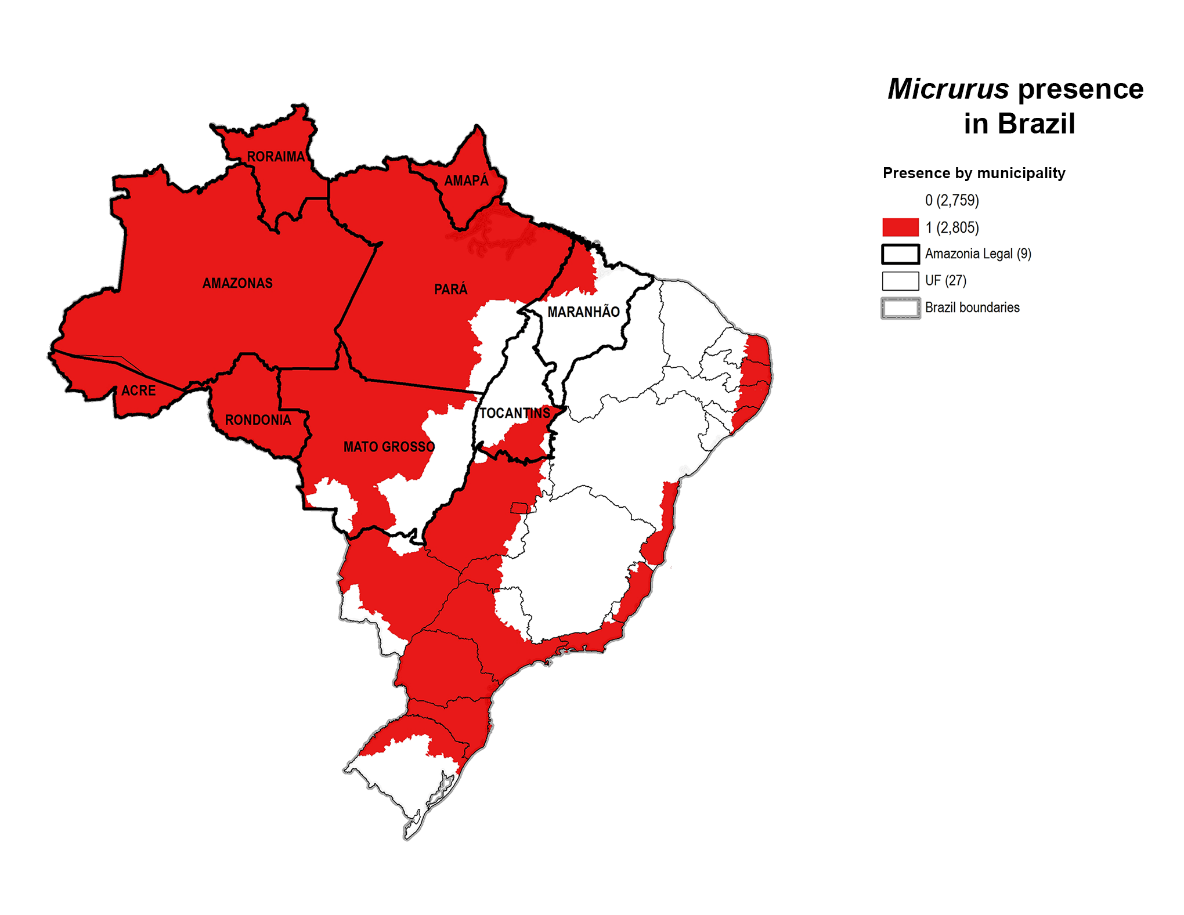


**Figure F.** Presence of the genus *Lachesis* by municipality in Brazil
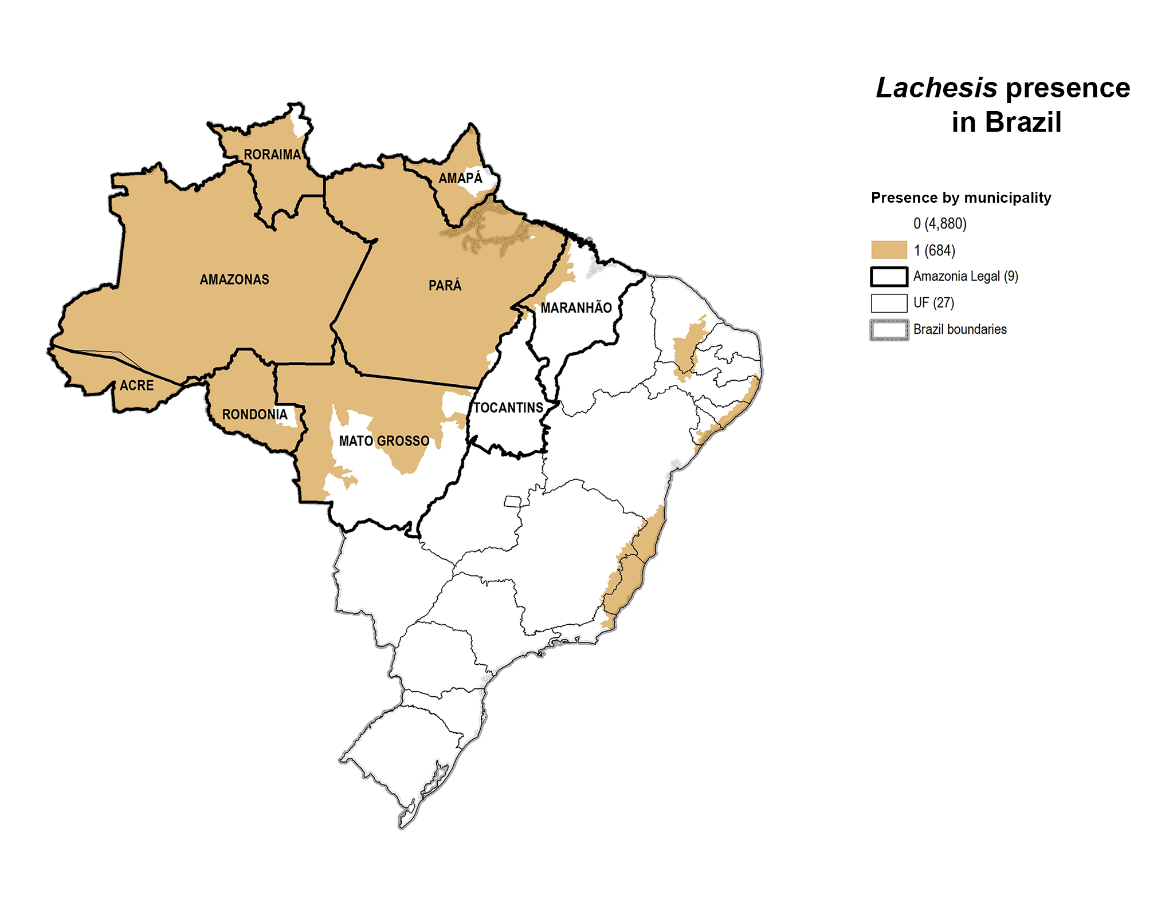

Supplement: S2 Text — Venomous snakes present in Brazil listed by World Health Organization (WHO), the Ministry of Health of Brazil, and the International Union for Conservation of Nature (IUCN). (DOCX) [file pntd.0009044.s002.docx]
