# Supplementary material for: Overview of snakebite in Brazil: Possible drivers and a tool for risk mapping
Source: PLoS Negl Trop Dis. 2021 Jan 29;15(1):e0009044. doi: 10.1371/journal.pntd.0009044 (PMC7875335; doi:10.1371/journal.pntd.0009044)
Supplement: S3 Text — Results of the descriptive, univariate, and multivariate analyses. (DOCX) [file pntd.0009044.s003.docx]

**Supporting information 3.** **Statistical Analysis**

**Descriptive analysis results**

**Table A.** Mean and standard deviation of the explanatory variables

| **Snakebite** | **Mean** | **Sd** | **Min** | **1st Qu.** | **Median** | **3rd Qu.** | **Max** |
| --- | --- | --- | --- | --- | --- | --- | --- |
| Count | 24·37 | 52·2 | 0 | 4 | 10 | 24 | 896 |
| Rate  (per 10^6) | 1339·5 | 1631·9 | 0 | 356·8 | 812·4 | 1710·7 | 30 786·3 |
| Tree loss (%) | 4·08 | 4·4 | 0 | 1·2 | 2·6 | 5·4 | 43·1 |
| Temperature | 22·24 | 3·0 | 13·5 | 19·8 | 22·5 | 24·7 | 27·8 |
| Precipitation | 1379·44 | 423·0 | 403·2 | 1141·1 | 1407·6 | 1625·6 | 3228·4 |
| Elevation | 337·54 | 256·5 | 0 | 116·0 | 295·0 | 529·3 | 1253·0 |
| GDP/capita (10^3^ RS) | 19·7 | 20·1 | 3·1 | 8·4 | 14·6 | 24·0 | 513·1 |
| Urbanization^4^ | 63·83 | 22·0 | 4·2 | 47·1 | 64·7 | 82·2 | 100 |
| Snake genus richness | 2·44 | 0·7 | 0 | 2 | 2 | 3 | 4 |
| Tree cover (km^2^) | 800·04 | 5082·3 | 0 | 26·9 | 78·8 | 250·2 | 150 961·9 |
| Spatial extent (km^2^) | 1528·11 | 5622·9 | 2·5 | 203·9 | 415·3 | 1027·9 | 159 522·9 |

**Table B.** Mean and standard deviation of the explanatory variables and p-value of t-test

| **Explanatory Variables** | **Mean (±sd)** | | | **P value ^3^** |
| --- | --- | --- | --- | --- |
|  | **Total** | **High incidence ^1^**  **(≥median rate^2^)** | **Low incidence ^1^**  **(< median rate^2^)** |  |
| Tree loss (%) | 4·08 ± 4·4 | 4·66±4·7 | 3·50±4·1 | <0·001 |
| Temperature | 22·4±3·0 | 22·35±2·9 | 22·13±3·0 | 0·004 |
| Precipitation | 1379·44±423·0 | 1470·24±449·2 | 1288·63±373·8 | <0·001 |
| Elevation | 337·54±256·5 | 336·8±252·8 | 338·32±260·2 | 0·819 |
| GDP/capita (10^3^ RS) | 19·7±20·1 | 18·40±17·7 | 20·91±22·2 | <0·001 |
| Urbanization^4^ | 63·83±22·0 | 58·15±20·2 | 69·52±22·4 | <0·001 |
| Snake genus richness | 2·44±0·7 | 2·50±0·7 | 2·38±0·7 | <0·001 |
| Tree cover (km^2^) | 800·04±5082·3 | 1429·00±7001·7 | 171·07±1361·7 | <0·001 |
| Spatial extent (km^2^) | 1528·11±5622·9 | 2381·12±7630·3 | 675·10±1889·1 | <0·001 |

^1^Snakebite rate (cumulative 2013–2017); ^2^ The median rate was 812·4 per 100 000
^3^ t-test; ^4^ proportion of urban population; ^5^ major habitat type (biome), categorize municipalities as tropical and non-tropical

**Table C.** Major habitat type (biome) and number of municipalities in that category

| **Biome** | **Categorization in our study** | **N** |
| --- | --- | --- |
| Deserts and xeric shrublands | Non-tropical | 1027 |
| Flooded grasslands | Non-tropical | 5 |
| Mangroves | Non-tropical | 50 |
| Tropical and subtropical dry broadleaf forests | Tropical | 90 |
| Tropical and subtropical grasslands, savannas, and shrublands | Tropical | 1189 |
| Tropical and subtropical moist broadleaf forest | Tropical | 3194 |
| No data |  | 9 |
| Sum |  | 5564 |

**Table D.** Major habitat type (biome) and number of snakebite cases in that category

| **Biome** | **Sum of bites** | **%** |
| --- | --- | --- |
| Deserts and xeric shrublands | 12 438 | 9·17 |
| Flooded grasslands | 560 | 0·41 |
| Mangroves | 2378 | 1·75 |
| Tropical and subtropical dry broadleaf forests | 1535 | 1·13 |
| Tropical and subtropical grasslands, savannas, and shrublands | 25 849 | 19·06 |
| Tropical and subtropical moist broadleaf forest | 92 146 | 67·96 |
| No data | 691 | 0·51 |
| Sum | 135 597 | 100 |

**Figure A. Correlation test (multi-collinearity check)**

- 1. **One-to-one correlation**


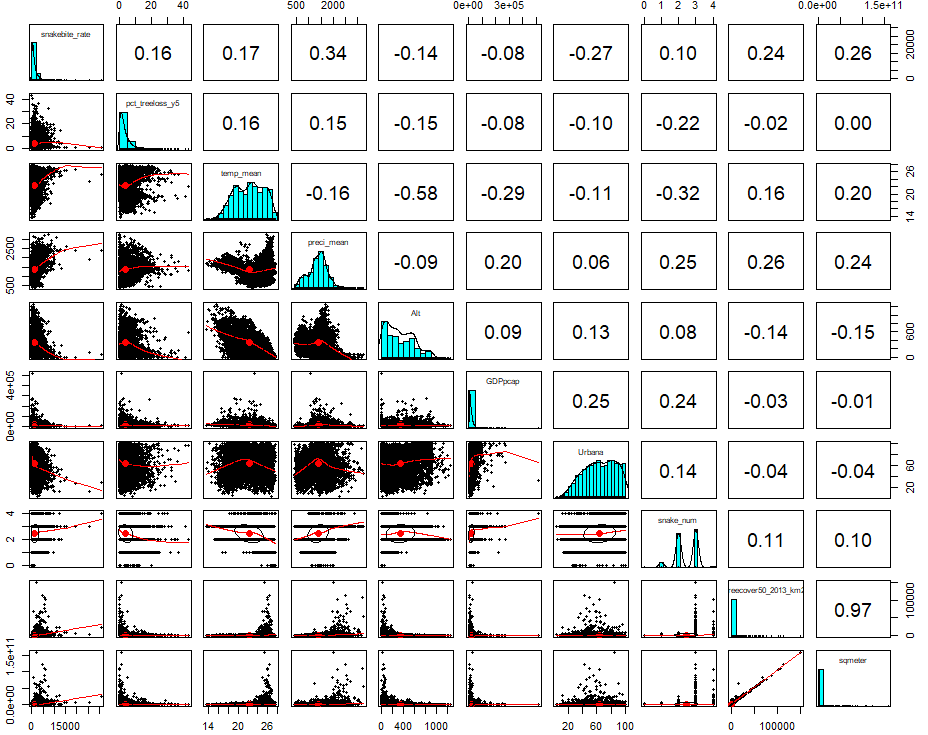


From the first row and column: 1) snakebite (rate), 2) forest loss (%) between 2013 and 2017, 3) mean temperature, 4) mean precipitation, 5) mean elevation, 6) GDP per capita, 7) urbanization, 8) snake genus richness, 9) tree cover in 2016, 10) spatial extent.

Obs. Because of higher correlation between tree cover and spatial extent (0·97), I decided to exclude spatial extent.

- 1. **VIF**

| **Explanatory variables** | **VIF** |
| --- | --- |
| Tree loss between 2013 and 2017 (%) | 1·32 |
| Temperature | 2·78 |
| Precipitation | 2·00 |
| Elevation | 2·09 |
| GDP per capita | 1·76 |
| Urbanization | 1·56 |
| Snake genus richness | 1·31 |
| Tree cover in 2013 | 1·23 |
| Biome (tropical/non-tropical) | 1·57 |

**Association (RR and 95% CI for IQR increase)**

**Table E. Univariate analysis**

| **Explanatory variables** | **Negative Binomial** |
| --- | --- |
| Tree loss between 2013 and 2017 | 1·201 (1·168–1·235) |
| Temperature | 1·426 (1·363–1·491) |
| Precipitation | 1·484 (1·444–1·526) |
| Elevation | 0·752 (0·721–0·785) |
| GDP per capita | 0·919 (0·901–0·937) |
| Urbanization | 0·524 (0·500–0·548) |
| Snake genus richness | 1·197 (1·151–1·244) |
| Tree cover in 2013 | 1·016 (1·014–1·019) |
| Biome (tropical/non-tropical) | 2·025 (1·886–2·173) |

*****RR for 1 IQR increase (except for the tropical variable)

*For biome variable, reference level was “non-tropical”. The RR is for tropical regions compared to non-tropical regions

**Table F. Multivariate analysis – four different combinations**

Combination 1. Full model (including tree cover and tropical)

| **Explanatory variables** | **Negative Binomial** |
| --- | --- |
| Tree loss between 2013 and 2017 | 1·066 (1·04–1·093) |
| Temperature | 1·647 (1·555–1·744) |
| Precipitation | 1·229 (1·183–1·277) |
| Elevation | 1·235 (1·176–1·297) |
| GDP per capita | 0·957 (0·939–0·975) |
| Urbanization | 0·507 (0·486–0·53) |
| Snake genus richness | 1·177 (1·132–1·224) |
| Tree cover in 2013 | 1·003 (1·002–1·004) |
| Biome (tropical/non-tropical) | 1·925 (1·755–2·11) |
| DIC | 41 232·2 |

*RR for 1 IQR increase (except for the tropical variable)

*For biome variable, reference level was “non-tropical”. The RR is for tropical regions compared to non-tropical regions

Combination 2. Excluding tree cover

| **Explanatory variables** | **Negative Binomial** |
| --- | --- |
| Tree loss between 2013 and 2017 | 1·06 (1·034–1·086) |
| Temperature | 1·693 (1·6–1·791) |
| Precipitation | 1·259 (1·214–1·307) |
| Elevation | 1·237 (1·178–1·299) |
| GDP per cap | 0·954 (0·937–0·973) |
| Urbanization | 0·504 (0·483–0·527) |
| Snake genus richness | 1·191 (1·146–1·239) |
| Biome (tropical/non-tropical) | 1·908 (1·739–2·092) |
| DIC | 41 251·9 |

*RR for 1 IQR increase (except for the tropical variable)

*For biome variable, reference level was “non-tropical”. The RR is for tropical regions compared to non-tropical regions

Combination 3. Excluding biome (tropical/non-tropical)

| **Explanatory variables** | **Negative Binomial** |
| --- | --- |
| Tree loss between 2013 and 2017 | 1·109 (1·081–1·137) |
| Temperature | 1·462 (1·382–1·547) |
| Precipitation | 1·448 (1·404–1·494) |
| Elevation | 1·234 (1·174–1·297) |
| GDP per capita | 0·958 (0·94–0·977) |
| Urbanization | 0·544 (0·521–0·568) |
| Snake genus richness | 1·181 (1·135–1·229) |
| Tree cover in 2013 | 1·002 (1·001–1·004) |
| DIC | 41 417·5 |

*RR for 1 IQR increase (except for the tropical variable)

Combination 4. Excluding tree cover and biome

| **Explanatory variables** | **Negative Binomial** |
| --- | --- |
| Tree loss between 2013 and 2017 | 1·103 (1·076–1·131) |
| Temperature | 1·499 (1·419–1·584) |
| Precipitation | 1·477 (1·434–1·521) |
| Elevation | 1·235 (1·175–1·299) |
| GDP per capita | 0·956 (0·938–0·975) |
| Urbanization | 0·541 (0·518–0·564) |
| Snake genus richness | 1·193 (1·147–1·241) |
| DIC | 41 431·6 |

*RR for 1 IQR increase (except for the tropical variable)
