# Supplementary material for: Overview of snakebite in Brazil: Possible drivers and a tool for risk mapping
Source: PLoS Negl Trop Dis. 2021 Jan 29;15(1):e0009044. doi: 10.1371/journal.pntd.0009044 (PMC7875335; doi:10.1371/journal.pntd.0009044)
Supplement: S4 Text — Results applied to Rio Grande do Sul state, flowchart to support decision-makers on snakebite risk by municipality in Brazil. (DOCX) [file pntd.0009044.s004.docx]

**Supporting information 4.** **Flowchart risk instrument results**

**Table A.** Quartiles rages for snakebite and rates per 100,000 population variables by municipality in Rio Grande do Sul

| **Variables** | **Min** | **1st Qu.** | **Median** | **3rd Qu.** | **Max** |
| --- | --- | --- | --- | --- | --- |
| Snakebites count | 0 | 1 | 5 | 11 | 118 |
| Rate (per 10^6) | 0 | 7.9 | 17.0 | 30.7 | 140.6 |

Other information for Rio Grande do Sul state

Population = total 11322895; median by municipality 5726

Median temperature = 18.8 C

Median precipitation = 1667.863 millimeters

Median elevation = 157 meters

Median GDP/Capita Reias = 28679.78

Median urbanization = 53.0%

Type of major habitat tropical or non-tropical in Rio Grande do Sul was all tropical

In the case of number of deaths, it was considered one death as the cutoff.

Median number of drivers by municipality in the state was two, considered many drivers more them two.

**Figure A.** Results applied to Rio Grande do Sul state, flowchart to support decision-makers on snakebite risk by municipality in Brazil


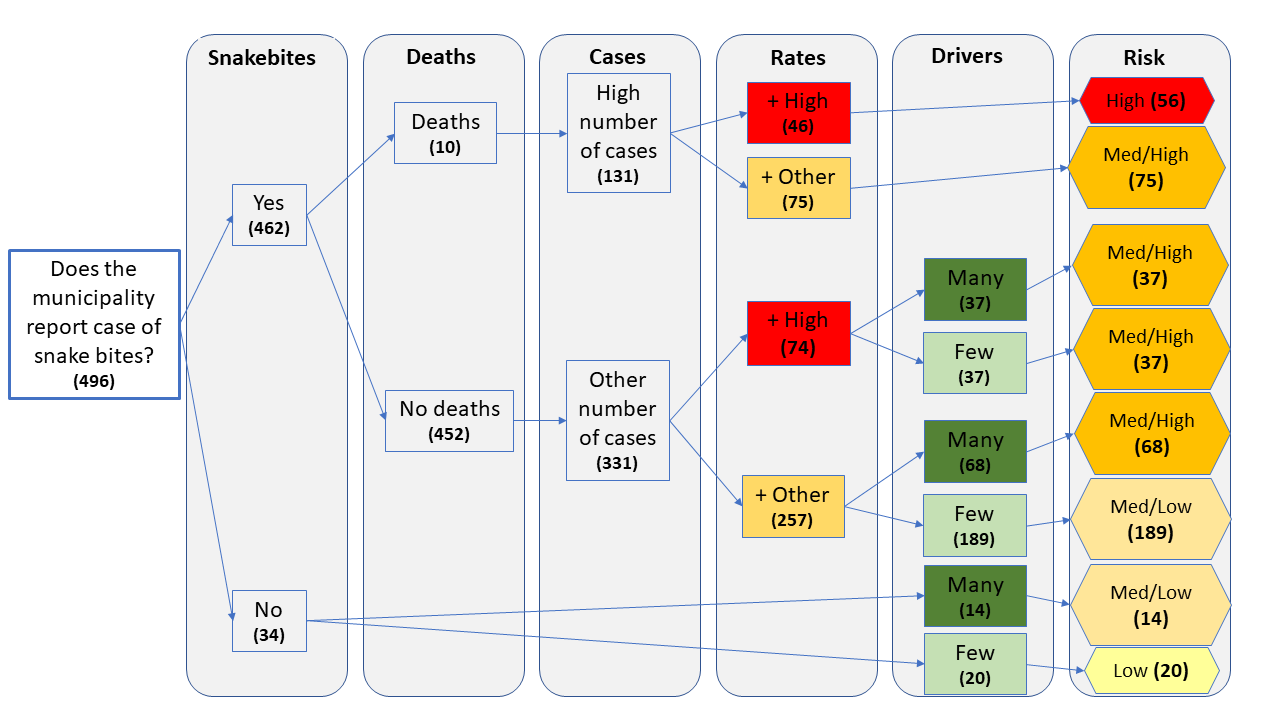


Number of cases of snakebite in the period: 4227

Number of municipalities with snakebite cases: 462/496

Number of municipalities with deaths by snakebite: 10/496

Number of municipalities with “high risk”: 56

Number of municipalities with “medium high risk”: 217

Number of municipalities with “medium low risk”: 203

Number of municipalities with “low risk”: 20
