# Supplementary material for: Overview of snakebite in Brazil: Possible drivers and a tool for risk mapping
Source: PLoS Negl Trop Dis. 2021 Jan 29;15(1):e0009044. doi: 10.1371/journal.pntd.0009044 (PMC7875335; doi:10.1371/journal.pntd.0009044)
Supplement: S5 Text — (DOCX) [file pntd.0009044.s005.docx]

**Supporting Information 5 Abstract in Portuguese - Resumo em português**

**Antecedentes** Envenenamentos por acidente com serpentes afeta quase 2,7 milhões de pessoas todos os anos em todo o mundo. No Brasil, acidentes por serpente soa notificados ao sistema de vigilância do Ministério da Saúde e recebida gratuitamente o soro. É necessário identificar áreas de maior risco para a distribuição de soro antiofídico e desenvolver ações preventivas. O objetivo deste estudo é fornecer uma visão geral da situação epidemiológica dos acidentes por serpente no Brasil e explorar possíveis fatores condutores; bem como criar uma ferramenta para apoiar os tomadores de decisão.

**Metodologia / Principais resultados** Um estudo do tipo ecológico foi realizado com dados por município (2013-2017). Partes do estudo: 1) Criar um banco de dados geocodificado e fazer uma análise descritiva e de cluster; 2) Análise estatística para medir a associação de acidentes por serpente e possíveis fatores ambientais e socioeconômicos; 3) Desenvolver um fluxograma para apoiar os tomadores de decisão e a aplicar esta ferramenta em um estado como exemplo.

Uma média anual de 27 120 casos de acidentes por serpente foi notificado no Brasil. Clusters de municípios com alto número acidentes por serpente são encontrados principalmente na Amazonia Legal. O modelo de regressão binomial negativa mostrou associação com casos de acidentes por serpente: e o tipo de habitat principal, tropical ou não tropical (RR = 1,92; IC95% = 1,75-2,10); temperatura (RR = 1,57; IC95% = 1,49-1,66); porcentagem de urbanização (RR = 0,50; IC95% = 0,48-0,53); precipitação (RR = 1,30; IC95% = 1,26-1,36); elevação (RR = 1,18; IC95% = 1,12–1,24); PIB per capita (RR = 0,96; IC95% = 0,94-0,98); uma relação mais fraca com a perda de floresta (RR = 1,04; IC95% = 1,02-1,06); e com abundância de serpentes venenosas (RR = 1,07; IC95% = 1,04-1,11). O DIC foi de 41 330,27. O estado em que o instrumento foi aplicado registrou 4227 acidentes por serpente no período. A maioria dos municípios foram considerados de risco médio e 56/496 como de alto risco, de acordo com a ferramenta criada.

**Conclusões / Importância** Os casos de acidentes por serpente são distribuídos por todo o país com a maior concentração na região da Amazônia Legal. Isso indica uma situação complexa, tanto para melhor compreender a associação de fatores ambientais e socioeconômicos com acidentes por serpente, quanto para a distribuição e manutenção de soro em áreas remotas. São necessárias pesquisas sobre tipos de soro antiofídico com vida útil mais longa, sem a necessidade de refrigeração.
