# Supplementary material for: Overview of snakebite in Brazil: Possible drivers and a tool for risk mapping
Source: PLoS Negl Trop Dis. 2021 Jan 29;15(1):e0009044. doi: 10.1371/journal.pntd.0009044 (PMC7875335; doi:10.1371/journal.pntd.0009044)
Supplement: S6 Text — (DOCX) [file pntd.0009044.s006.docx]

**Supporting Information 6. Abstract in Spanish – Resumen en español**

**Antecedentes** El envenenamiento por mordedura de serpiente afecta a casi 2.7 millones de personas cada año en todo el mundo. En Brasil, los accidentes por serpiente son informados al sistema de vigilancia del Ministerio de Salud y reciben el anti-veneno de forma gratuita. Es necesario identificar áreas de mayor riesgo para su distribución y desarrollar acciones preventivas. El objetivo de este estudio es proporcionar una visión general de la situación epidemiológica del envenenamiento por mordedura de serpiente en Brasil y explorar posibles factores conductores; así como crear una herramienta para apoyar a los tomadores de decisiones.

**Metodología/ Hallazgos principales** Se realizó un estudio de tipo ecológico utilizando datos por municipio (2013-2017). Partes del estudio: 1) Crear una base de datos geocodificada y realizar un análisis descriptivo y de conglomerados; 2) Análisis estadístico para medir la asociación de la mordedura de serpiente y los posibles factores ambientales y socioeconómicos; 3) Desarrollo un diagrama de flujo para apoyar a los tomadores de decisiones y la aplicación de esta herramienta en un estado como ejemplo. Se informó un promedio de 27 120 casos de accidentes por serpiente por año en Brasil. Los grupos de municipios con gran número de mordeduras de serpientes se encuentran principalmente en la Amazon Legal. El modelo de regresión binomial negativa mostró asociación con el recuento de casos de mordeduras de serpientes: el tipo de hábitat principal, tropical o no tropical (RR = 1.92; CI95% = 1.75–2.10); temperatura (RR = 1.57; CI95% = 1.49–1.66); porcentaje de urbanización (RR = 0.50; IC95% = 0.48–0.53); precipitación (RR = 1.30; CI95% = 1.26–1.36); elevación (RR = 1.18; IC95% = 1.12–1.24); PIB per cápita (RR = 0,96; IC95% = 0,94–0,98); una relación más débil con la pérdida de florestas (RR = 1.04; CI95% = 1.02–1.06); y con abundancia de serpientes venenosas (RR = 1.07; IC95% = 1.04–1.11). El DIC fue 41 330,27. El estado donde se aplicó el instrumento reportó 4227 mordeduras de serpiente en el período. La mayoría de los municipios fueron considerados de riesgo medio y 56/496 como de alto riesgo según la herramienta creada.

**Conclusiones / Importancia** Los casos de mordedura de serpiente se distribuyen en todo el país con la mayor concentración en la región de la Amazonía Legal. Esto crea una situación compleja tanto para una mejor comprensión de la asociación de factores ambientales y socioeconómicos con los accidentes por serpientes como para la distribución y mantenimiento de anti-veneno a áreas remotas. Se necesita investigación sobre los tipos de anti-veneno con una vida útil más larga sin necesidad de refrigeración.
